# Rheumatoid arthritis patients display B-cell dysregulation already in the naïve repertoire consistent with defects in B-cell tolerance

**DOI:** 10.1038/s41598-019-56279-0

**Published:** 2019-12-27

**Authors:** Yan Wang, Katy A. Lloyd, Ioannis Melas, Diana Zhou, Radha Thyagarajan, Joakim Lindqvist, Monika Hansson, Anna Svärd, Linda Mathsson-Alm, Alf Kastbom, Karin Lundberg, Lars Klareskog, Anca I. Catrina, Stephen Rapecki, Vivianne Malmström, Caroline Grönwall

**Affiliations:** 1 Division of Rheumatology, Department of Medicine Solna, Karolinska Institutet, Karolinska University Hospital, Stockholm, Sweden; 2 UCB Pharma, Slough, UK; 3 Division of Rheumatology, Department of Clinical and Experimental Medicine, Linköping University, Linköping, Sweden; 4 Center for Clinical Research Dalarna, Uppsala University, Uppsala, Sweden; 5 Thermo Fisher Scientific and Uppsala University, Uppsala, Sweden

**Keywords:** Autoimmunity, B cells, Translational immunology, Rheumatic diseases, Rheumatoid arthritis, Antibodies, Adaptive immunity

## Abstract

B cells are postulated to be central in seropositive rheumatoid arthritis (RA). Here, we use exploratory mass cytometry (n = 23) and next-generation sequencing (n = 19) to study B-cell repertoire shifts in RA patients. Expression of several B-cell markers were significantly different in ACPA^+^ RA compared to healthy controls, including an increase in HLA-DR across subsets, CD22 in clusters of IgM^+^ B cells and CD11c in IgA^+^ memory. Moreover, both IgA^+^ and IgG^+^ double negative (IgD^−^ CD27^−^) CD11c^+^ B cells were increased in ACPA^+^ RA, and there was a trend for elevation in a CXCR5/CCR6^high^ transitional B-cell cluster. In the RA BCR repertoire, there were significant differences in subclass distribution and, notably, the frequency of VH with low somatic hypermutation (SHM) was strikingly higher, especially in IgG1 (p < 0.0001). Furthermore, both ACPA^+^ and ACPA^−^ RA patients had significantly higher total serum IgA and IgM compared to controls, based on serology of larger cohorts (n = 3494 IgA; n = 397 IgM). The observed elevated Ig-levels, distortion in IgM^+^ B cells, increase in double negative B cells, change in B-cell markers, and elevation of unmutated IgG^+^ B cells suggests defects in B-cell tolerance in RA. This may represent an underlying cause of increased polyreactivity and autoimmunity in RA.

## Introduction

Rheumatoid arthritis is a systemic inflammatory disease with a complex pathogenesis, involving multiple cellular pathways in potentially different phases of the disease. One of the hallmarks is the presence of anti-citrullinated protein autoantibodies (ACPA) and rheumatoid factor autoantibodies which defines the seropositive subset of RA (reviewed in^1^). This autoreactivity is already present in the pre-clinical phase of disease that precedes development of chronic joint inflammation^2,3^.

While it was previously debated if ACPA IgG solely should be considered an important biomarker or an active promoter of disease, mounting evidence from studies of purified or monoclonal autoantibodies now point towards ACPA indeed having a direct pathogenic functionality by contributing to inflammation, fibroblast and osteoclast activity, as well as potentially mediating pain mechanisms^4–9^. However, other autoreactive antibodies may also share some of these features^10,11^. Consequently, this emphasizes a central role for the adaptive immune system and B cells in the initiation and progression of pathogenesis in RA. Furthermore, B-cell clonal expansion has been detected in RA, and IgA^+^ plasmablast and dominant clone elevations can be detected in pre-RA individuals with ACPA autoimmunity^12–14^. Recent findings have also revealed interesting molecular characteristics of the immunoglobulin anti-citrulline immune response such as high somatic hypermutation levels, introduced Fab-glycosylation sites in variable regions, and selective cross-reactivity to multiple citrullinated antigens by recognition of linear consensus epitopes which sometimes extends to other post-translational modifications^15–23^. These observations may reflect a unique B-cell selection process in RA with high B-cell activity and ACPA^+^ B cells undergoing sequential germinal center cycles.

Rheumatoid factor (RF) immunoglobulins on the other hand, carry relatively modest somatic hypermutation numbers^24,25^. RF^+^ B cells also show a distinctly different transcriptional profile compared to ACPA^+^ B cells, with more innate-like pathways active^25^. RFs are composed primarily of IgM isotype, although IgA and IgG are also present, and they could be postulated to be part of a (possibly dysregulated) feedback system for clearance of immune complexes, similar to what has been postulated for the IgM natural antibody repertoire. Natural IgM are produced by specialized innate-like B cells, spontaneously expressed from birth in a T-cell independent manner, and are germline encoded (reviewed in^26,27^). While these IgM have anti-inflammatory properties and have been hypothesized to be beneficial due to their role in clearance of dead cells and modified biomolecules^28–30^, the B cells may also act as a pool of polyreactive and self-reactive cells that could get engaged during break-of tolerance and lead to T-cell dependent pathogenic autoreactivity. They may especially contribute to inflammation and autoimmunity if class-switched to IgG. Interestingly, our previous study showed that RA patients have increased levels of natural IgM to oxidation-associated autoantigens^11^, highlighting the potential of IgM reactivity in RA.

In this study we use two platforms, mass cytometry and repertoire sequencing, to exploratorily investigate B-cell phenotype and B-cell receptor (BCR) characteristics in RA. Our observations demonstrate significant shifts in the B-cell populations, especially in naïve B cells, as well as distortions in the immunoglobulin repertoire with increased unmutated IgG, that are consistent with defective B-cell regulation in RA. By using an in-depth B-cell focused methodology, we can start to decipher the underlying B-cell repertoire changes in RA in detail.

## Results

### RA B cells have elevated expression of cell surface markers, including HLA-DR and CD11c

**Figure 1:**
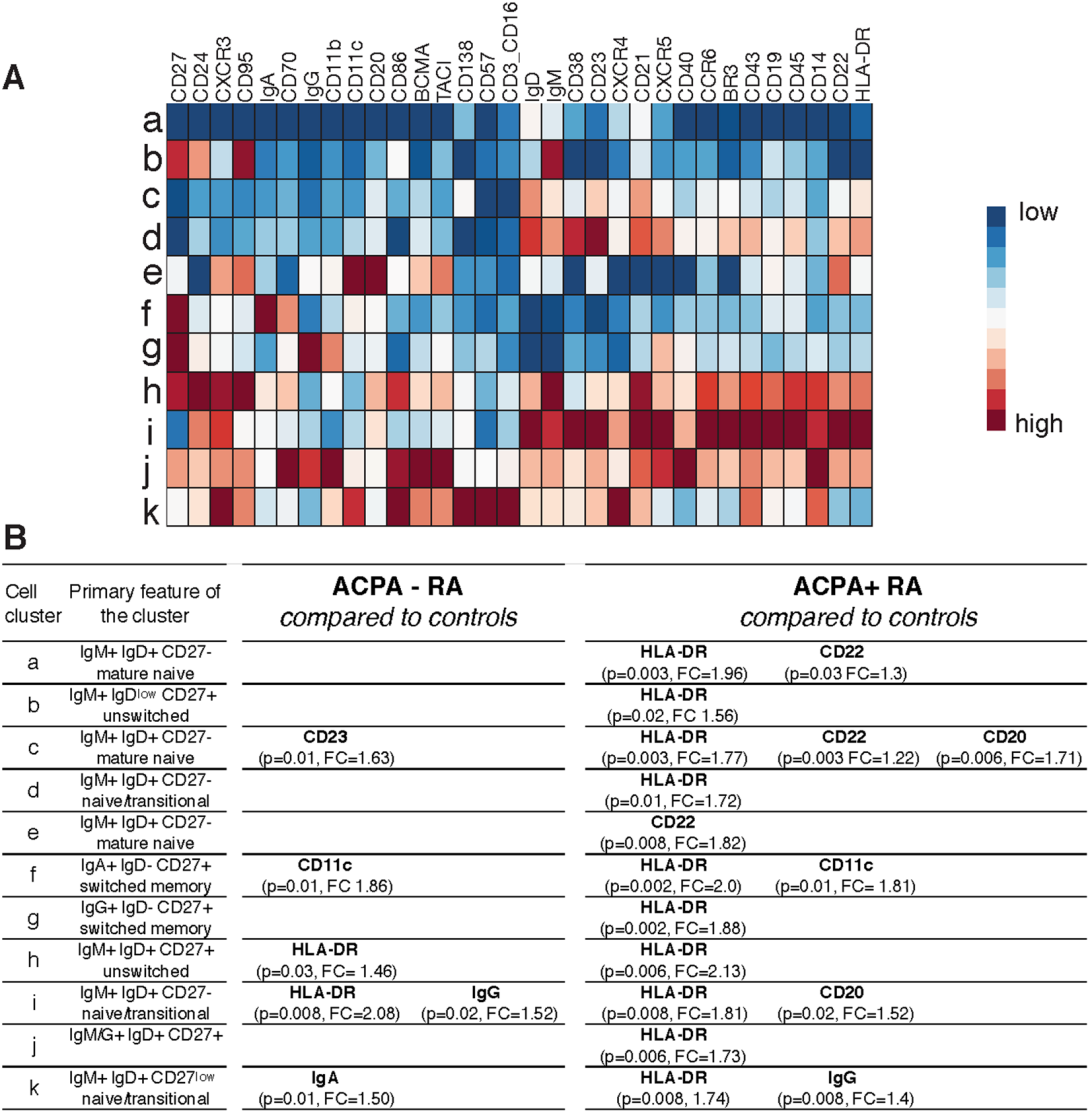
Differential expression of surface markers in rheumatoid arthritis determined by mass cytometry. The figure shows result from mass cytometry analysis of purified peripheral B cells from nine ACPA^+^ RA patients, seven ACPA^−^ RA patients, and seven healthy controls with a 35-marker panel (Supplemental Table 3). (**A**) Normalized expression profiles for the B-cell clusters (a-k) with differential expression of surface markers between RA and controls. The heatmap is visualizing the expression levels of surface markers for the B cell clusters in a color scale from red (high expression) to blue (low expression) compared to the other clusters. (**B**) Significant markers, P-values from t-test and fold changes (FC) are presented in the right panel comparing ACPA^+^ or ACPA^−^ RA with healthy controls.

We studied circulating B cells by mass cytometry in nine ACPA^+^ and seven ACPA^−^ RA patients, compared to seven matched healthy controls. Deep B-cell phenotyping was achieved through a B-cell enrichment strategy and a 35-channel phenotypic panel. Two different analysis strategies were used in parallel; (i) analyzing the differential expression of surface markers within cell clusters, and (ii) evaluating the cell counts in cell clusters for shifts in population sizes. By analyzing the data in these unbiased ways, we firstly identified 11 cell clusters (designated a-k) displaying differential expression of surface markers between the groups (heat map Fig. 1A, Supplemental Fig. 2). The most prominent differentially expressed marker was HLA-DR, which was increased in ACPA^+^ RA compared to controls across B-cell clusters, including clusters that represent both naïve (clusters a, c, d: IgD^+^CD27^−^) and memory (clusters f & g: IgD^−^CD27^+^) B-cell populations, irrespectively of the Ig isotype (Fig. 1B, fold change = 1.6–2.1). This elevated expression in ACPA^+^ RA was present also in cell clusters with overall relatively low HLA-DR expression. ACPA^−^ RA patients, on the other hand, had increased HLA-DR expression compared to controls only in certain B-cell clusters. Another significant finding was that CD22 (Siglec-2) was increased in ACPA^+^ RA in several IgD^+^ IgM^+^ CD27^−^ cell clusters (Fig. 1A,C). Interestingly, we observed significantly increased CD11c in both ACPA^+^ and ACPA^−^ RA in an IgA^+^ switched memory phenotype (IgD^−^CD27^+^) B-cell cluster (Fig. 1B; cluster f). Performing manual gating confirmed that HLA-DR was significantly increased in ACPA^+^ RA also when analyzing all B cells (p = 0.02) (Supplemental Fig. 3). We could also see trends for a general increase of B-cell CD11c expression that was borderline significant, but this was detected primarily in ACPA^−^ RA (p = 0.05).

### RA patients show shifts in naïve and memory B-cell sub-populations

**Figure 2:**
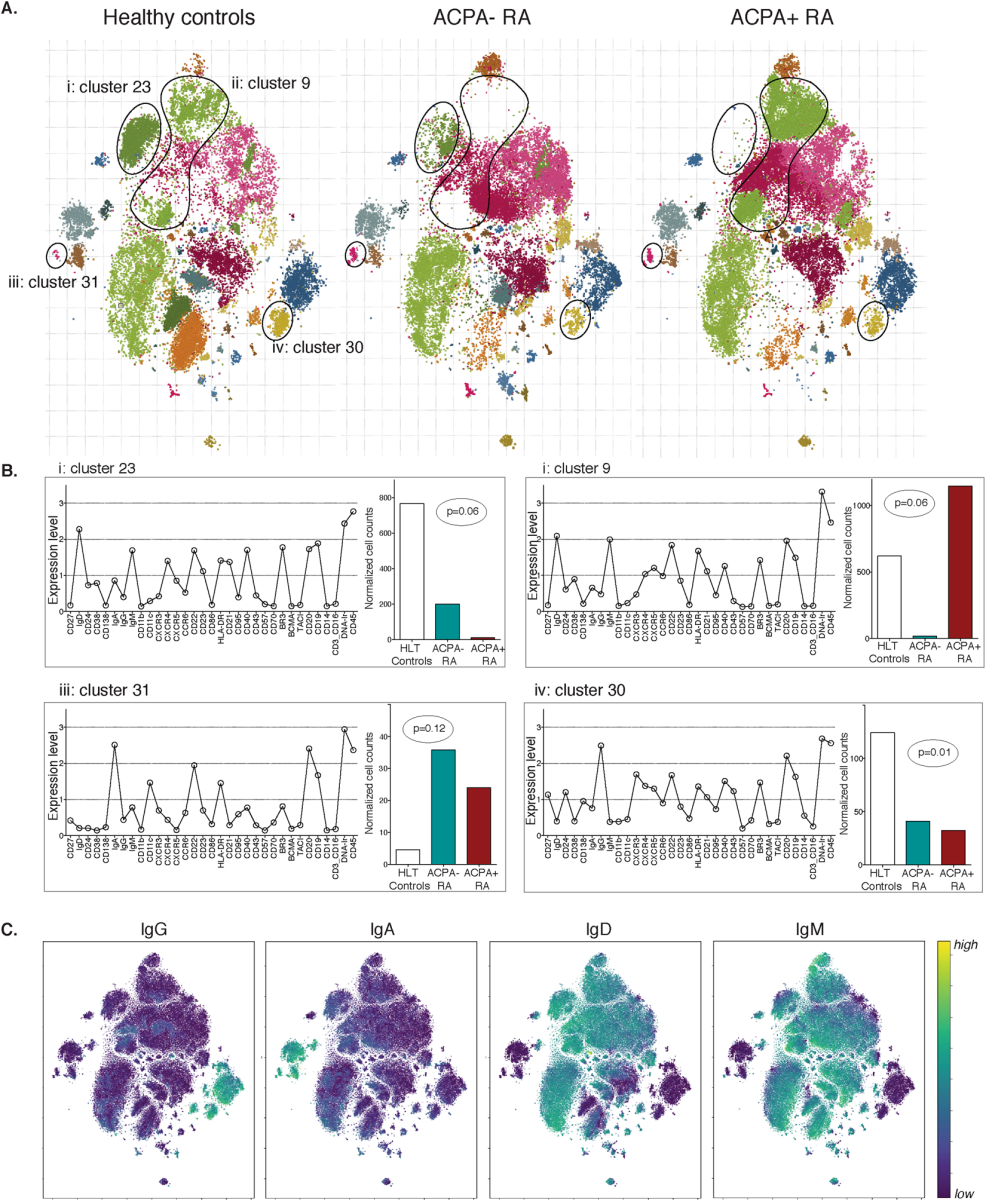
In-depth B-cell phenotype in rheumatoid arthritis by mass cytometry. The figure shows results from mass cytometry analysis of purified peripheral B cells from seven ACPA^−^ RA patients, nine ACPA^+^ RA patients and seven healthy controls with a 35-marker panel (Supplemental Table 3). Data was analyzed to evaluate cell count differences between tSNE identified cell clusters (**A**) tSNE visualization of clustering for comparing cell counts in ACPA^−^ RA, ACPA^+^ RA, and controls. Identified cell clusters with significant or borderline significant ANOVA differences between the three groups, after adjusting for sex, age and sample processing date, are highlighted in (**A)** (i–iv) and presented in more detail in (**B**) showing relative expression of the different markers in the B-cell clusters and the normalized cell counts in the three groups. (**C**) tSNE visualization highlighting the immunoglobulin isotype expression levels. Cell counts were normalized in the analysis.

Next, we performed cluster analysis and dimensionality reduction via tSNE to elucidate the pattern of B-cell distribution, and identified several different B-cell clusters with distinct phenotypic profiles (Fig. 2 and Supplemental Figs. 4, 5, 7) that varied across our three subject groups. Strikingly, we could detect no less than 21 IgM clusters and 10 class-switched B-cell clusters. Of the class-switched clusters three IgA^+^, four IgG^+^ and three with mixed IgM/IgA and IgM/IgG clusters were observed. The variation within the ‘naïve’ B-cell population was unexpected, but clearly seen when depicting IgD and IgM expression in a separate tSNE (Fig. 2C). Since our preliminary analysis suggested that cell preparation on different days (including MACS separation and staining) introduced some bias, we adjusted all analyses for cell preparation date, acquisition date, sex and age to account for these potential confounder effects. The identified B-cell clusters could to a large extent be explained by expected populations (mature naïve B cell, memory B cells, transitional B cells etc.) using traditional markers (CD27, IgD, CD24, CD38)^31^, whereas expression profiles of chemokine receptors (CXCR3-5, CCR6) and CD43 seemed to discriminate the B cells into a larger number of sub-populations (Supplemental Figs. 6 and 8). In addition, the detection of three isotypes (IgM, IgA and IgG) provided further information about the B-cell phenotypes. While many different IgM^+^ B cell clusters were observed, the different IgA^+^ and IgG^+^ class-switched clusters showed great homology, except for expressing either IgA or IgG (Supplemental Fig. 6). Notably, there were also a couple of CD27^+^ sub-populations with co-expression of IgM and IgG or IgA which were observed in a few individuals and that may represent intermediate class-switched phenotypes.

We used analysis of variance (ANOVA) to investigate statistical differences in relative cell counts between the three groups; healthy controls, ACPA^−^ and ACPA^+^ RA. Among the class-switched B-cell clusters, we could see a significant difference (p = 0.01) in an IgG^+^CD27^+^CD24^+^CD43^+^ B-cell sub-population with lower cells numbers in both ACPA^−^ and ACPA^+^ RA (cluster 30, Fig. 2, Supplemental Figs. 4 and 5, Table 1). We could also detect a trend for an increase in a small IgA^+^ double negative (IgD^−^CD27^−^) CD11c^+^ cell cluster in both ACPA^+^ and ACPA^−^ RA (cluster 31; p = 0.12). Similarly, when performing a sub-analysis comparing only ACPA^+^ RA with controls (Table 1), we could see that the number of cells in both IgG^+^ and IgA^+^ double negative (IgD^−^CD27^−^) B-cell clusters were significantly increased in ACPA^+^ RA (cluster 21 p = 0.01 and cluster 31 p = 0.03, respectively). Interestingly, the most striking differences between the three groups were observed in cell clusters representing IgM^+^IgD^+^CD27^−^CD38^int^ CD24^int^ mature-naïve or transitional immature B-cell sub-populations with a decrease in one B-cell sub-population (cluster 23) and an increase in another IgM^+^ B-cell cluster (cluster 9) in ACPA^+^ RA (Fig. 2, Supplemental Figs. 4 and 6). While the statistical differences between the patient groups were just below significance (p = 0.06), the stringency of the analysis and the rather large cell count differences, nevertheless implicate these findings as biologically relevant and interesting. The two B-cell populations were to a large extent similar and we would most likely not have been able to discriminate between them by flow cytometry. The main differences seem to be a shift in chemokine receptor expression with slightly higher CCR6 and CXCR5 expression and lower CXCR4 and CD40 in cell cluster 9 that was expanded in ACPA^+^ RA (Supplemental Fig. 9). In accordance, we could not see any difference between groups using only manual gating of the separate chemokine markers in either all B cells or IgM^+^ B cells (data not shown).

**Table 1:** Statistical analysis of cell counts in different RA B-cell clusters by mass cytometry.

B-cell cluster	Primary feature of the cluster	Fold difference* ACPA -	Fold difference* ACPA+	ANOVA comparing three groups (p-value)	ANOVA comparing ACPA+ with controls (p-value)
23	IgM+ IgD+ CD27- (naive/transitional)	0.26	0.01	0.06	*NS* > *0.2*
9	IgM+ IgD+ CD27- (naive/transitional)	0.03	1.85	0.06	*NS* > *0.2*
6	IgM+ IgD+ CD27- (mature naive)	1.85	2.26	*NS* > *0.2*	0.04
26	IgM+ IgD+ CD27- CD38+ (naive/transitional)	3.35	2.41	*NS* > *0.2*	0.06
30	IgG+ IgD- CD27+ (IgG switched memory)	0.33	0.26	0.01	0.03
20	IgA+ IgD- CD27+ (IgA switched memory)	0.83	0.59	*NS* > *0.2*	0.04
21	IgG+ IgD- CD27- (IgG double neg)	8.70	5.81	*NS* > *0.2*	0.01
31	IgA+ IgD- CD27- (IgA double neg)	5.33	7.96	0.12	0.03

*For normalized cell count values compared to healthy controls.

When investigating the overall BCR isotype distribution in the B cells, we could not see any significant difference in the number of IgM and IgG positive cells. However, there was a trend for decreased percentage of circulating IgA positive cells in ACPA^+^ RA (Supplemental Fig. 10).

We conclude from the phenotype studies that shifts in the circulating B-cell population, as well as significant differences in B-cell surface marker expression, can be observed in RA patients. To further depict B-cell differences, we next performed in-depth studies of the BCR transcripts in ACPA^+^ RA.

### BCR repertoire studies in RA reveal differences in class-switching and increased number of B cells expressing unmutated IgG

**Figure 3:**
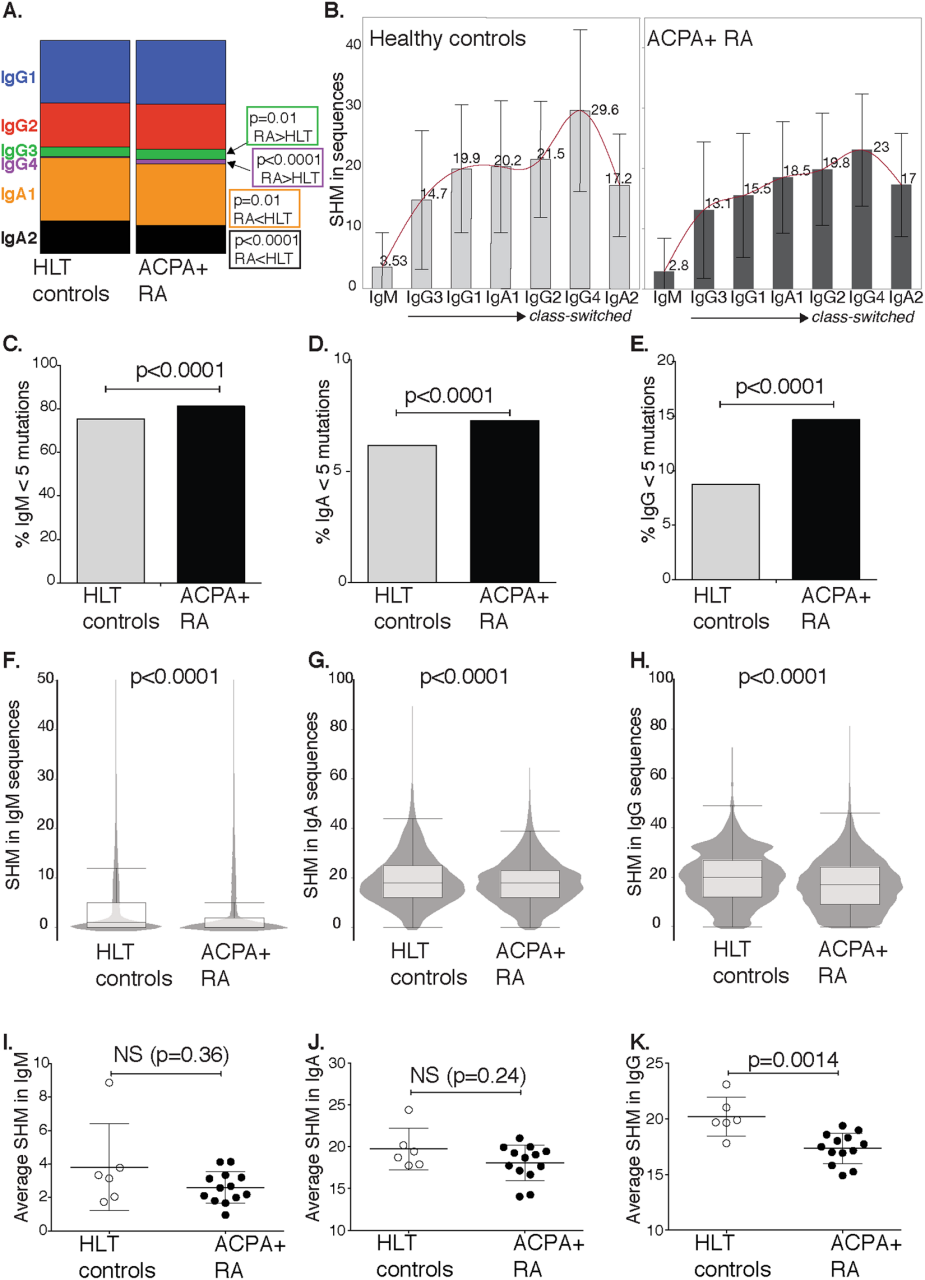
BCR repertoires in rheumatoid arthritis by next generation sequencing. Data are presented from Illumina Miseq sequencing of heavy and light chain immunoglobulin variable region transcripts from peripheral B cells in 13 ACPA^+^ RA patients and six healthy controls (HLT). (**A**) Distribution of subclass expression among class-switched (IgG and IgA) sequences from healthy controls compared to sequences from RA patients. (**B**) Somatic hypermutation (SHM) level distributed per isotype and subclass. Frequency of unmuted sequences (<5 mismatches) among IgM (**C**), IgA (**D**) and IgG (**E**). SHM levels in IgM (**F**,**I**), IgA (**G**,**J**), and IgG (**H**,**K**). The (F–H) panels are showing result from pooled sequences (Turkey outliner box blots with an overlay violin plots), while the I–K panel are showing the average SHM in sequences from individual subjects. The level of SHM was determined by number of mismatches in comparison to the closest germline sequence in the IMGT database. P-values are presented from chi-square with Yates’ correction or Mann-Whitney analysis.

To explore BCR sequence repertoires, we utilized a multiplex PCR sequencing approach to obtain both heavy and light chain libraries from circulating B cells from 13 ACPA^+^ RA patients and six healthy controls. In line with the mass cytometry experiments, we observed a significantly lower frequency of circulating IgA^+^ B cells in ACPA^+^ RA among class-switched cells compared to the controls (Fig. 3A; 42% vs 45% of class-switched, p < 0.0001). This was seen in both IgA1 and IgA2 sequences. In contrast, there was slightly higher frequency of IgG3 (Fig. 3A, 4.5% vs 4.9%, p = 0.01) in RA. Notably, IgG4 sequences were rare in the healthy controls but readily detected in the RA patients (0.56% vs 2.1%, p < 0.001).

The most striking differences were observed when analyzing the somatic hypermutation (SHM) levels in the BCR variable regions. The levels of SHM were consistently significantly lower in RA immunoglobulin sequences than in sequences from the control subjects (Fig. 3). We could detect lower levels in all IgG subclasses as well as in IgA and IgM, although the most dramatic differences were seen in IgG and particularly in IgG1 sequences (Fig. 3, Table 2, Supplemental Fig. 11), with an average number of 15.2 SHM compared to the closest germline in RA and 19.9 SHM in sequences from healthy individuals (p < 0.0001). Consequently, the analysis of sequences from the RA patients also demonstrated a higher frequency of B cells expressing unmutated immunoglobulins, defined by <5 mismatches towards IMGT germline sequences in RA compared to healthy controls, in the IgM (81.2% vs 75.4%), IgA (7.3% vs 6.2%) and IgG (14.7% vs 8.7%) sequences (Fig. 3C–E). Of note, this indicates a 1.7-fold increase of unmutated IgG in RA. Naturally, the immunoglobulin repertoires vary between individuals (Supplemental Fig. 12). Yet, even with the limited number of subjects in this study, the average IgG SHM numbers were remarkably consistent within the groups and were significantly different between the groups (Fig. 3K, p = 0.0014). The light chains, lacking isotype origin, did not show as much difference in mutation level, although kappa chain sequences displayed similar decrease in SHM in ACPA^+^ RA (Supplemental Fig. 13, Table 2).

**Table 2:** RA patients have significantly lower BCR SHM levels.

BCR	Heathy control sequences*	RA sequences*	p-value**
IgM	3.5 ± 5.5 (n = 87330)	2.8 ± 5.8 (n = 154170)	p < 0.0001
IgG	20.1 ± 10.6 (n = 15890)	17.1 ± 10.3 (n = 33927)	p < 0.0001
IgA	19.2 ± 10.3 (n = 12981)	18.1 ± 9.1 (n = 24640)	p < 0.0001
IgG1	19.8 ± 10.6 (n = 8441)	15.5 ± 10.2 (n = 17433)	p < 0.0001
IgG2	21.5 ± 9.6 (n = 5991)	19.8 ± 9.3 (n = 12408)	p < 0.0001
IgG3	14.7 ± 11.5 (n = 1297)	13.1 ± 11.3 (n = 2859)	p < 0.0001
IgG4	29.6 ± 13.4 (n = 161)	23.0 ± 9.3 (n = 1227)	p < 0.0001
all VL	10.9 ± 9.7 (n = 28511)	10.4 ± 8.8 (n = 87515)	p = 0.0042
lambda	10.5 ± 9.7 (n = 14940)	10.0 ± 8.4 (n = 38202)	NS
kappa	11.3 ± 9.8 (n = 13571)	10.7 ± 9.1 (n = 49313)	p < 0.0001

*Average SHM/mismatches compared to closest germline sequence among analyzed sequences ±SD.

**P-value from Mann-Whitney analysis.

All RA patients were CCP2 positive.

We utilized an analysis strategy based on annotation of unique sequences, considering the whole variable region to define the BCR sequence rather than CDR3-based clonotypes. However, when instead using clonotype assignment (defined by VDJ and CDR3) we could utilize the number of unique molecular identifiers (UMIs) for a certain clonotype as a measure of expansion (or RNA transcript load). We concluded that while expansions in low mutated clones (<5 SHM) could be seen in a couple of RA patients, expansions were more commonly detected in highly mutated clonotypes (>15 SHM; Supplemental Fig. 14C). Therefore, the RA-associated increase of unmutated IgG does not seem to be derived from a few expanded clonotypes, but are instead likely to be a more general polyclonal phenomenon.

In summary, ACPA^+^ RA is associated with increased class-switching to IgG without SHM, implying that class-switching is occurring without affinity maturation and germinal center reactions.

### Seropositive RA patients have increased serum IgA and IgM levels

**Figure 4:**
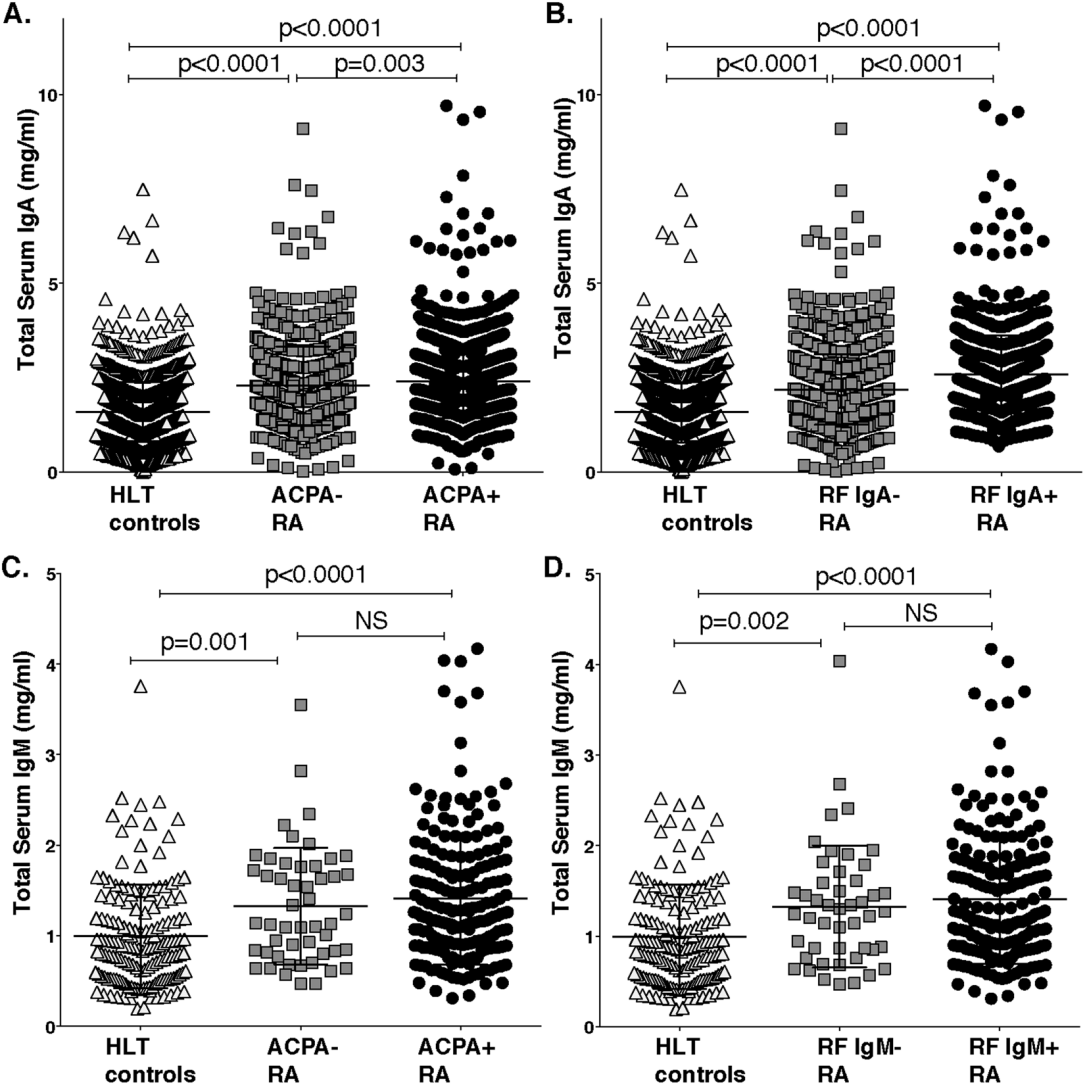
Increased serum levels of IgA and IgM in rheumatoid arthritis. (**A**,**B**) The figure is showing serum IgA levels in 1979 RA patients and 1300 population controls determined by reversed-phase microarray methodology. (**C**,**D)** Serum IgM levels were determined by sandwich ELISA in 243 RA patients and 154 population-based controls. (**A**) IgA levels in controls compared to 1250 ACPA^+^ RA patients and 729 ACPA^−^ RA patients. (**B**) Serum IgA levels in 1141 RF IgA^−^ and 831 RF IgA^+^ RA patients. (**C**) IgM levels in controls compared to 193 ACPA^+^ RA patients and 50 ACPA^−^ RA patients. (**D**) IgM levels in controls compared to 196 RF IgM^+^ RA patients and 47 RF IgM^−^ RA patients. P-values are presented from Kruskal-Wallis analysis with Dunn’s correction of multiple comparisons. ACPA positivity was determined by IgG anti-CCP2 test (Euro Diagnostica).

While circulating IgA cells were lower in both mass cytometry and BCR sequence analysis, when we analyzed total IgA levels in 2194 RA patients 1300 population controls (Fig. 4, Supplemental Table 10), we found that IgA levels were significantly elevated in RA compared to controls (2.32 ± 1.0 [median 2.21] vs 1.58 ± 0.80 mg/ml [median 1.51], p < 0.0001). ACPA^−^ individuals also had increased IgA levels compared to controls, yet the levels were slightly higher in ACPA^+^ RA (ACPA^−^ n = 730, 2.28 ± 1.0 mg/ml [median 2.15]; ACPA^+^ n = 1250, 2.39 ± 0.97 mg/ml [median 2.25], p = 0.003). The elevation in ACPA^−^ RA patients could not be explained by RF positivity, although the levels were slightly higher in RF IgA positive or CCP2 IgA positive patients (Fig. 4 and Supplemental Fig. 15). When dichotomizing the patients into seropositive and seronegative based on all available autoantibody tests (RF- IgM, IgA, and IgG; CCP2- IgG and IgA) we could detect a significant elevation in serum IgA in both the seronegative group (n = 465, 2.21 ± 0.96 mg/ml [median 2.1], p < 0.0001) and in the seropositive group (n = 1517, 2.39 ± 1.0 mg/ml [median 2.2], p < 0.0001) compared to controls (Supplemental Fig. 15).

To further evaluate the differences detected in RA naïve and unmutated B cells by the phenotype and repertoire studies, we performed a serological screening for total IgM levels in a subset of the cohort with 193 ACPA^+^ RA patients, 50 ACPA^−^ RA and 154 population-based controls (Fig. 4, Supplemental Table 10). We found that total IgM levels were significantly higher in ACPA^+^ RA compare to controls (1.41 ± 0.72 mg/ml vs 0.99 ± 0.57 mg/ml, p < 0.0001). Interestingly, there was also a significant yet numerically smaller increase in IgM levels in ACPA^−^ RA compared to controls (1.32 ± 0.64 mg/ml vs 0.99 ± 0.57 mg/ml, p = 0.002). There was no direct correlation between total IgM levels and RF IgM levels in the RA patients (data not shown), and also RF IgM^−^ RA patients displayed higher IgM levels than the population controls (Fig. 4). In addition, there was no significant difference in RA patients that were HLA shared epitope positive compared to those that were negative (data not shown). Interestingly, in both RA and controls IgM levels were higher in females than males, whilst IgA levels instead were higher in males (data not shown). Yet, the IgM and IgA level differences between RA and healthy controls remained significant in regression analysis after adjusting for age, sex, smoking and HLA shared epitope (p < 0.0001; Supplemental Table 10). Hence, the increased serum Ig levels seems to be primarily associated with RA disease, although higher in antibody-positive disease.

## Discussion

We highlight here a central role of B cells in RA showing shifts in the B-cell phenotype, BCR repertoire, and overall immunoglobulin titers in RA. While to a large extent exploratory, our study emphasizes the importance of the adaptive immune system and B cells in RA, and indicates that B-cell distortion in RA may be part of the underlying cause of the autoreactivity profile leading to disease.

Through in-depth B-cell phenotyping, we observed higher expression of HLA-DR across B-cell populations in seropositive RA consistent with more activated B cells. We could also detect higher levels of CD22, which is a co-receptor of the BCR with a reactivity to α2,6-linked sialic acids^32^ (reviewed in^33^), in several IgM^+^ B-cell populations in ACPA^+^ RA. While CD22 is negative regulator of BCR, its roles on different B cell subset is complex and expression can be increased on activated B cell after certain stimuli.

Interestingly, while we did see a trend for increased CD11c in all RA B cells, the statistically significant elevation of CD11c was primarily seen in an IgA^+^ switched memory cell population. CD11c is associated with the so-called atypical age- or autoimmunity-associated B cells (ABCs)^34^, but it should be noted that the CD11c differential expression, although increased in RA, was still overall relatively moderate in the IgA cluster compared to the cell clusters with highest CD11c. Furthermore, phenotypically these IgA^+^ cells did not display other features of ABCs (CD11c^high^ CD27^−^ CD21^−^). Yet when analyzing cell counts, we could indeed also see an increase in two clusters of CD11c^high^ CD27^−^ CD21^low^ CD20^high^ IgA^+^ or IgG^+^ B cells in the RA patients that may be ABC-related. It is certainly possible that these two observations are connected. Previous studies have also shown that older women with RA have an expansion of a CD11c^+^ CD27^+^ IgG^+^ B-cell population, although the studies did not include IgA^35^. Of note, CD11c^+^CD21^low^ B cells have been suggested to be less responsive to BCR and CD40 stimulation, and upregulate inhibitory markers^36^. This is interesting in light of the fact that both our phenotypic and BCR repertoire investigations demonstrate that RA patients have lower numbers of IgA^+^ B cells in the circulation, potentially suggesting that numbers as well as the functionality of existing IgA^+^ B cells are altered in RA. Nevertheless, we still observed significantly increased IgA levels in both seropositive and seronegative RA. Interestingly, a previous report has also shown that IgA^+^ plasmablasts are elevated in individuals at-risk of developing RA^13^. While our experimental setup was not optimal for plasmablast detection, we could detect increased expansion of IgA^+^ BCR clones in a few RA individuals that could potentially originate from plasmablasts (Supplemental Fig. 14). Break-of-tolerance in RA is thought to start outside the joint, most likely in the mucosal interface (reviewed in^1^) where IgA is abundantly expressed. Different pathways have been proposed, including gut microbiome dysbiosis, epitope mimicking in periodontitis and citrullination in the lungs as triggering factors^37–42^. In this context, investigation of IgA and IgA^+^ B cells may provide important insights about the pathophysiology of RA. However, there are of course differences in IgA1 and IgA2, both in terms of expression, IgA1 being the predominant form in serum, and in Ig-locus location and class-switching, with no class-switching from mucosal IgA2 to IgG. Since our studies were limited to peripheral B cells, they may not reflect IgA expression in the tissue, and we cannot conclude if differences depend on exhaustion, migration or more fundamental changes.

Some of the most striking B-cell shifts were detected in the IgM^+^ B cells. We observed large numerical cell count differences between in two CD27^−^IgD^+^IgM^+^CD24^int^ CD38^int^ B-cell clusters, with one being lower in RA and one being higher in RA, and the RA elevated cluster exhibiting higher CXCR5 and CCR6 and lower CD40 and CXCR4. While the tSNE cell clusters do not represent distinct B-cell populations, they may in this case illustrate different activation levels or migration states. In mice, the chemokine receptor CXCR5 and CCR6 are expressed on transitional T2 B cells and mature recirculating follicular B cells^43^. The receptors and their ligand CXCL13 and CCL20 have been suggested to be important for B-cell migration into the inflamed synovium in human arthritis, and CCR6 has been reported to be increased in peripheral B cells from arthritis patients^44^. In regards to naïve B-cell populations, previous studies have shown that new-onset and very early RA patients had an increased frequency of naïve (IgD^+^CD27^−^) B cells and lower levels of unswitched (IgD^+^CD27^+^) cells, supporting a distortion in naïve or transitional B-cell populations^45,46^. This is consistent with the hypothesis that RA patients have defects in tolerance checkpoints leading to accumulation of polyreactive cells in the mature naïve B-cell compartment^47^. Notably, the naïve B-cell population can be more affected by disease duration and DMARD treatment^45,46^. However, as discussed above, we could still detect trends in the naïve B cells although the majority of patients included in this study had received synthetic DMARDs or TNF blockade and had different disease duration. Besides the distortion in naïve B cells, we also detected elevation in the double negative (IgD^−^CD27^−^) B-cell subset that was most evident when comparing the ACPA^+^ RA patients with controls. An increase in double negative cells in RA has also previously been reported^48^. Importantly, the double negative population have gained increasing interest in the field of autoimmunity, especially since in SLE they have been connected to autoimmunity, originate from naïve cells, and represent pre-plasmablasts^49^. A small study have also suggested that CD27^−^ IgG^+^ B cells have lower VH SHM levels than CD27^+^ IgG^+^ B cells^50^.

Next-generation sequencing is providing new opportunities for insights in B-cell repertoires in autoimmune disease (discussed in^51^) and additional investigations are required to fully understand how different B-cell clones are related. Previous efforts have focused on clonal expansion in early or pre-RA^14,52,53^. With our methodology and limited data set, we could not detect any significant difference between peripheral clonal expansion in RA compared to controls. However, the use of cryopreserved samples may limit our ability to detect plasmablast that may not survive the thawing process. Also, with bulk methodology it is generally not possible to separate clonal expansions and presence of plasmablasts with substantially higher Ig-transcript levels.

In our BCR repertoire studies, we were surprised to see that RA patients consistently displayed lower lever of SHM compared to controls, and this was observed also in sub-analysis of different isotypes and subclasses. The subclasses followed expected levels of mutations in association with the immunoglobulin locus and the most frequent class-switch order (IgG3 < IgG1 < IgG2 < IgG4)^54^. Nevertheless, the differences seem to be most distinctive in the IgG1 subclass. SHM are a strong indication of germinal center (GC) reactions and affinity maturation, and consequently we hypothesize that RA B cells exhibit increased extrafollicular class-switching that occurs outside of the GC responses. A previous meta-analysis did not find significant differences in RA compared to control, yet the studies had access to a limited number of sequences^55,56^. Notably, the specific anti-citrullinated protein autoantibodies are hallmark autoreactivity in RA and through single-cell efforts have been shown to generally feature very high level of SHM^15,21,22^. Hence, the unmutated B cells are unlikely to directly reflect circulating autoreactive ACPA^+^ B cells. Rheumatoid factor B cells may on the other hand have a different origin than ACPA^+^ B cells (discussed in^57^), although they also display SHM and evidence of antigen-selection but at a more modest level^24,25^. In conclusion, the expressed germline-encoded BCRs may not be ACPA or RF, but are instead likely to be low affinity or polyreactive^47^ clones, suggesting that RA patients may have an elevation of polyreactive IgG^+^ B cells. We hypothesize that these cells instead may provide a baseline pool for expansion of potential autoreactive clones. Yet, both our B-cell phenotype and repertoire investigations were cross-sectional and included a limited number of individuals. Hence, more extensive and longitudinal studies are merited to further evaluate the RA B-cell dysregulation. It would be especially interesting to follow the B-cell repertoire in association with disease progression in ACPA^+^ at-risk individuals.

In relation to the low mutation IgG, IgG autoantibodies to malondialdehyde (MDA) post-translational modifications are also significantly increased in RA and have been hypothesized to be associated with inflammation^11,58^. These IgG anti-MDA can, in contrast to ACPA, be expressed by germline-encoded variable regions and associated with the natural antibody repertoire. Besides the IgG isotype, we have also previously reported that natural IgM toward the prototypic antigens MDA and phosphorylcholine (PC) are significantly increased in RA compared to controls^11^. A large proportion of circulating IgM may be considered to come from the spontaneously expressed natural IgM pool. Hence, the overall increased serum level of IgM that we report here within could partly reflect the increased specific natural IgM (e.g anti-MDA) that we have previously reported^11^. Of course, the differences in actual IgM levels were modest and well within the range of variation in the general population^59^. Yet, the data further supports that there are significant differences in the B-cell repertoire, perhaps especially in the naïve and innate-like B-cell repertoire.

Interestingly, we could also observe differences in B-cell phenotype and increased IgM and IgA total immunoglobulin serum levels in ACPA seronegative RA patients. The seronegative RA patient subset, without detectable ACPA or RF autoantibodies, remains an enigma. Without the HLA class II genetic association and distinct environmental risk factors seen in seropositive RA, this is generally considered a disease with different pathophysiology, where autoimmunity supposedly would play a less important role. Hence, we did not expect to see any changes related to the adaptive immune system. However, several reports have suggested that at least a subset of seronegative RA express different autoantibodies, including ACPA fine-specificities, that cannot be detected with the classical clinical CCP/RF assays^60–63^. Therefore, seronegative RA is a heterogeneous group and further studies are needed to understand if there indeed is an autoimmunity component, presumably distinct from ACPA^+^ RA, present in a subset or a larger proportion of these patients.

In conclusion, our data demonstrates that RA patients have distortions in naïve B cells as well as lower IgA^+^ B cells, increased double negative class-switched memory B cells, increased class-switched B cells with low SHM, and elevated IgM and IgA serum levels. Altogether, this strongly supports that RA is associated with increased natural antibodies and defects in tolerance mechanisms, leading to increases in naïve polyreactive B cells which class-switch in a T-cell independent way without somatic hypermutations and may accumulate in the in double negative pre-plasmablast subset. When the polyreactive cells get activated and engaged into germinal center responses, they may acquire somatic hypermutations and stronger autoreactivity. More extensive studies are now required to understand if these distinct B-cell distortions are a cause or consequence of the autoimmune disease and what role B cells play in different phases of RA progression.

## Methods

### Patients and controls

Blood samples were collected from RA patients and healthy age-matched blood donors without any rheumatic disease (for details see Supplemental Tables 1 and 2). Peripheral blood mononuclear cells (PBMC) were isolated from heparinized whole blood by Ficoll-Paque (GE healthcare Life Science) and cryopreserved until analysis. Serum samples from 2194 RA patients and 1300 population controls from the Epidemiological Investigation of Rheumatoid Arthritis (EIRA) study^64^ were screened for total IgA levels. Analysis of IgM, IgG and IgA rheumatoid factor (RF) was performed on a Phadia ImmunoCap 250 instrument according to the manufacturer**’**s instructions. IgA anti-CCP2 was measured by EliA™ (Phadia AB, Uppsala, Sweden) with a Phadia 250 instrument (Phadia AB) as previously reported^65^. Total serum IgA levels were measured by reversed-phase microarray methodology as previously reported^66^. IgM levels were further screened in a subset of 243 RA patients and 154 population-based controls. Total IgM levels were screened by an in-house sandwich ELISA. Briefly, high-binding half area ELISA plates (Corning) were coated with goat anti-human IgM (μ-chain specific, Jackson Immunoresearch), blocked with 3% BSA in PBS, and serum samples analyzed at 1:50 000 and 1:150 000 dilution in 1% BSA in PBS using mouse anti-human IgM HRP (Southern Biotech) detection and purified polyclonal IgM (Sigma Aldrich) as a reference. CCP positivity was determined by the CCPlus clinical test (Euro Diagnostica), and confirmed with CCP3 Quanta Lite IgG (Inova Diagnostics) and/or by antigen microarray^67^ for patients included in mass cytometry and NGS studies. Of the 2194 RA patients in the IgA serology study, 2166 had available HLA genotype data and 1979 had available IgG anti-CCP2. Patients included in the NGS and mass cytometry studies could have received DMARD treatment including methotrexate and TNF blockade but not any biological treatment directed towards B or T cells (i.e rituximab, abatacept). The EIRA patients included in serological studies had not received any DMARD treatment at the time of sample collection. The study was performed in compliance with the declaration of Helsinki and was approved by the regional ethical review board in Stockholm. All experiments were performed according to good clinical practice and good laboratory practice. All study subjects gave informed consent to participate. All RA patients fulfilled the 2010 ACR classification criteria^68^.

### Mass cytometry analysis of B cells

Cryopreserved PBMC samples were thawed in warm RPMI medium with 10% FBS, benzonase treated (Sigma Aldrich) and washed two times. Four samples were processed at the same time, randomizing RA samples with healthy controls. B cells were isolated by negative separation using the human B-cell isolation kit II (Miltenyi Biotec) and QuadroMACs separator. B cells were counted (yields varied between 0.2–1.9 × 10^6^), washed, and stained in RT at 10^7^ cells/ml with Cisplatin Cell-ID 194Pt in PBS for cell viability (1 μl:8 ml). The staining was quenched with Cell Staining Media (CSM; PBS, 1% BSA, 0.02% sodium azide) and after washing the cells were taken up in 200 μl staining mix and incubated on ice 45 min. A custom designed staining panel with 32 surface markers plus one dump channel (combining CD3/CD16) and two viability/singlets controls (Cell-ID Cisplatin and DNA Intercalator) were utilized (see Supplemental Table 3). All antibodies were in-house labeled using MaxPar labeling kits (Fluidigm Corporation) at UCB Pharma following the manufacturer’s instructions, titrated and evaluated for binding to human PBMCs and/or control cell lines before use. The antibodies were used either at 1 μl, 2 μl or 5 μl/test (Supplemental Table 3). After staining the cells were taken up in 200 μl fixation buffer (4% formaldehyde) with DNA intercalator Cell-ID (Fluidigm) at 1:5000 dilution and stored at 4 °C until acquisition. Thereafter, samples were washed with CSM followed by MaxPar water (Fluidigm), and EQ calibration beads (Fluidigm) were applied for correction of instrument variation and samples were acquired on a CyTOF Helios (CyTOF2 upgrade) mass cytometry system (Fluidigm). Cell samples were processed and stained on four different days, batched, and acquired on two separate days. Equal distribution between groups were ensured in both sample processing and acquisition.

### Mass cytometry data analysis

The raw data was normalized based on the EQ beads, EQ bead data was subsequently removed, and files from each donor concatenated. The mass cytometry data was thereafter pre-processed in FlowJo software (Tree Star) by gating on singlets, live cells, and B cells (CD19^+^ CD3/CD16^−^) and the generated files were used in all down-stream analysis. On average 194000 events of enriched B-cells were acquired per patient (Range: 32000–600000) and on average 115000 live single cell events were obtained (Range: 6080–449000). Gating strategy is shown in Supplemental Fig. 1. The mass cytometry data was parsed, processed and normalized using the flowCore and flowStats packages in R (www.r-project.org) using logicle transformation. Thereafter, single cell data from all samples were pooled together, and clustered via flowSOM^69^ in R to identify distinct cell populations. To visualize single cell events as a scatterplot, dimensionality reduction was first performed using an R implementation of tSNE (https://cran.r-project.org/web/packages/Rtsne/index.html). Finally, the number of cells in each cluster/population attributed to the subject groups (healthy controls, ACPA^−^ RA and ACPA^+^ RA) was calculated to identify over-represented patient groups across clusters. This was performed using ANOVA to account for batch effects and confounding factors. The analysis included age, sex, cell preparation date and acquisition date as confounding variables. The Benjamini Hochberg correction for multiple testing was applied. Very small cell populations or populations that could not clearly be determined to be B cells were excluded from the data presentation. Additionally, differential regulation of markers across groups was calculated via t-test. Briefly, we examined each marker separately, assumed its activation follows a normal distribution with characteristics depended on the patient group it was measured in. Then we compared the distribution originating from either ACPA^+^ RA or ACPA^−^ RA versus the distribution originating from population controls using a one tailed t-test. Details of cluster profiles are presented in Supplemental Figs. 2 and 4–6.

### Preparation of NGS BCR libraries

After thawing, 2.5 × 10^6^ PBMC were lysed with 500 μl cell lysis buffer (100 mM Tris-HCl, pH 7.5, 10 mM EDTA, 500 mM LiCl, 1% LiDS and 5 mM DTT), frozen on dry ice and stored at −80 °C until use. Before the extraction, each sample was split into two equal aliquots, for IgG/IgA and IgM/IgK/IgL analysis, respectively. Reverse primers for each group were added to the cell lysate at a concentration of 500 nM in total, followed by the addition of 50 μl Oligo(dT) beads (NEB). The samples were incubated at 65 °C for 10 minutes, room temperature for 15 minutes, and ice for at least 15 minutes. The mRNA was purified following the manufacturers’ protocol, but with an extra washing step with 100 μl PCR buffer (20 mM Tris-HCl, 50 mM KCl, 3 mM MgCl_2_, pH 7.5). Thereafter, the washed beads were resuspended with 60 μl Superscript III RT-PCR Master Mix without any primer oligos. The first strand cDNA was synthesized by incubating at 55 °C for 50 minutes then 70 °C for 15 minutes. Five microliters of the RT-PCR products with beads was used as template in the following PCR amplification. Heavy or light chain PCR reactions were set up with HotStarTaq® Plus DNA Polymerase (Qiagen) with CoralLoad PCR Buffer and 0.1 μM heavy chain variable region primer pool (for IgG/IgA and IgM) or 0.05 μM of each light chain variable region primer pool (for IgK/IgL) were used as forward primers, and 0.2 μM i7 adapter primer as reverse primer. PCR reactions were performed for 1 cycle of 94 °C for 5 minutes, followed by 35 cycles of 94 °C for 30 seconds, 54 °C for 30 seconds and 72 °C for 1 minutes, and a final extension of 72 °C for 10 minutes. For light chains, a secondary PCR using 10 μl of the initial PCR product was performed to attach the i5 Illumina adapter at 5′ end using CoralLoad PCR Buffer, 0.4 μM of i5 adapter primer (annealing to an extension adaptor included in the first PCR primers) and i7 adapter primer as reverse primer. PCR reaction were performed for 1 cycle of 94 °C for 5 minutes, followed by 6 cycles of 94 °C for 30 seconds, 58 °C for 30 seconds and 72 °C for 1 minute, and a final extension of 72 °C for 10 minutes. All PCR products from above were purified with MiniElute Gel Extraction Kit (Qiagen) following the manufacturer’s guidelines. The concentrations of the purified PCR products were adjusted to 4 ng/μl. A PCR for attaching the complete Illumina adapters with index were performed with HotStarTaq® Plus DNA Polymerase with CoralLoad PCR Buffer, 5 μl of the genomic DNA (20 ng) were used as template and 0.2 μM of complete Illumina adapters with i5 or i7 index were used as forward and reverse primers. PCR reactions were performed for 1 cycle of 94 °C for 5 minutes, followed by 10 cycles of 94 °C for 30 seconds, 58 °C for 30 seconds and 72 °C for 1 minutes, and a final extension of 72 °C for 10 minutes. The PCR products were subsequently purified by gel extraction as above, concentration was determined and final libraries were mixed and submitted for Illumina MiSeq 2 × 300 bp sequencing. All primers used in the library preparation are reported in Supplemental Tables 4–8.

### BCR repertoire analysis

For sequences generated from mRNA, low quality sequences were filtered away and duplicated sequences combined using pRESTO^70^. The 5′ end primers were identified from R1 read directions, while 3′ end primers and universal molecular identifier (UMI) were identified from the R2 reads of a sequence. After a multiple sequence alignment of the sequences with same UMI, a consensus sequence was generated for each UMI and replaced all the sequences within the same UMI group. The read pairs were assembled into a full sequence. Duplicated identical sequences with different UMI were then identified and collapsed into a unique sequence. The number of duplicated sequences within UMI group were calculated and UMI groups with low duplication number were excluded (<2 duplications). All remaining sequences were annotated based on sample ID and combined into one file (final sequence numbers are available in Supplemental Table 9). The IgG and IgA subclasses were assigned using pRESTO. Thereafter, all the sequences were submitted to IMGT HighV-quest for immunoglobulin gene assignment. Statistical analysis was performed using JMP (SAS Institute) and Prism (GraphPad Software). Chi-square with Yates’ correction was used for comparing frequencies and Mann-Whitney analysis for distributions.

## Supplementary information

Supplementary Information

## Data Availability

The datasets generated during and/or analyzed during the current study are available from the corresponding author on reasonable request.
