# Supplementary material for: Rheumatoid arthritis patients display B-cell dysregulation already in the naïve repertoire consistent with defects in B-cell tolerance
Source: Sci Rep. 2019 Dec 27;9:19995. doi: 10.1038/s41598-019-56279-0 (PMC6934703; doi:10.1038/s41598-019-56279-0)
Supplement: Supplementary file 1 — Supplementary Information [file 41598_2019_56279_MOESM1_ESM.pdf]

Supplemental Table 1. Subjects included in mass cytometry study

Number of Subject	Groups*	Age	Sex	Disease duration	Steroid	csDMARD	TNFi
7	ACPA negative RA	Average: 63<br>Range: 30-75	4 F; 3 M	Average: 7.4y<br>Range : 0-27y			
				Treatments for each individual:	-	-	-
					PRED	LEF	-
					-	MTX	-
					-	MTX	ETAN
					-	-	-
					#	#	-
					-	MTX	-
9	ACPA positive RA	Average: 55<br>Range: 36-76	8 F; 1 M	Average: 13.5y<br>Range: 1-29y			
				Treatments for each individual:	PRED	MTX	INFL
					-	LEF	-
					PRED	MTX	-
					-	SAL	-
					-	MTX	INFL
					-	SAL	
					-	MTX	GOLI
					-	-	-
					-	MTX	ADAL
7	Healthy controls€	Average: 51<br>Range: 32-59	6 F; 1 M	N/A			

* ACPA positivity was determined by CCP2 from clinical record and verified with IgG anti-CCP3 (Inova Diagnostics) and ACPA fine-specificity antigen-microarray assay

PRED-prednisolone; LEF-leflunomide; SAL-salazopyrin (Sulfasalazin) MTX-methotrexate;
ETAN-etanercept TNF blockade; INFL-infliximab TNF blockade;
GOLI-golimumab TNF blockade; ADAL-adalimumab TNF blockade
#previous SAL and PRED

€ The control subjects were healthy individuals without any rheumatic disease. No information about other possible inflammatory conditions was available. An effort was made to match the controls by age and sex with RA patients as a group.

Supplemental Table 2. Subjects included in NGS study

Number of Subject	Groups*	Age	Sex
13	ACPA positive RA	Average: 61<br>Range: 30-80	9F<br>4M
6	Healthy controls€	Average: 58<br>Range: 52-67	4F<br>2M

* ACPA positivity was determined by CCP2 test (Euro Diagnostica) and verified by ACPA fine-specificity antigen microarray assay.

€ The control subjects were healthy individuals without any rheumatic disease. No information about other possible inflammatory conditions was available. An effort was made to match the controls by age and sex with RA patients as a group.

Supplemental Table 3. Mass cytometry panel

ANTIGEN	TAG*	CLONE	COMPANY	CAT. NO	Staining
CD21	141Pr	Bu32	Biolegend	354902	2µl/test
CD20	142Nd	2H7	Biolegend	302343	5µl/test
CD86	144Nd	IT2.2	Biolegend	305435	5µl/test
CD27	146Nd	O323	Biolegend	302839	5µl/test
IgM	147Sm	MHM-88	Biolegend	314527	2µl/test
CD95 (Fas)	148Nd	DX2	Biolegend	305631	2µl/test
CD19	149Sm	HIB19	Biolegend	302247	5µl/test
CD183 (CXCR3)	150Nd	G025H7	Biolegend	353733	5µl/test
CD138	151Eu	DL-101	Biolegend	306523	5µl/test
CD184 (CXCR4)	152Sm	12GS	Biolegend	306523	5µl/test
CD3	154Sm	UCHT1	Biolegend	300437	1µl/test
CD16	154Sm	3G8	Biolegend	302051	1µl/test
IgA	155Gd	Polyclonal	Jackson Immunoresearch	109-005-011	5µl/test
CD45	156Gd	5B1	Miltenyi Biotec	130-108-020	5µl/test
CD23	158Gd	EBVCS-5	Biolegend	338502	5µl/test
IgD	159Tb	IA6-2	Biolegend	348235	2µl/test
CD43	160Gd	CD43-10G7	Biolegend	343202	5µl/test
CD11b	161Dy	ICRF44	Biolegend	301337	2µl/test
CD269 (BCMA)	162Dy	19F2	Biolegend	357502	5µl/test
CD22	163Dy	HIB22	Biolegend	302511	5µl/test
TACI	164Dy	1A1	Biolegend	311902	5µl/test
CD57	165Ho	HCD57	Biolegend	322325	2µl/test
CD14	166Er	M5E2	Biolegend	301843	5µl/test
CD196 (CCR6)	167Er	G034E3	Biolegend	353427	5µl/test
CD24	168Er	ML5	Biolegend	311127	5µl/test
HLA-DR	169Tm	L243	Biolegend	307651	1µl/test
CD268 (BR3)	170Er	11C1	Biolegend	316902	5µl/test
CD70	171Yb	113-16	Biolegend	355102	5µl/test
CD40	172Yb	5C3	Biolegend	334325	2µl/test
IgG	173Yb	Polyclonal	Jackson Immunoresearch	109-005-098	2µl/test
CD185 (CXCR5)	174Yb	J252D4	Biolegend	356902	2µl/test
CD11c	175Lu	Bu15	Biolegend	337221	2µl/test
CD38	176Yb	HIT2	Biolegend	303535	2µl/test
DNA (Cell-ID™ Intercalator-Ir)	191Ir/193Ir	N/A	Fluidigm	201192A	1µl/test
Cisplatin (Cell-ID™)	194Pt	N/A	Fluidigm	201194	1µl/test

*All antibodies were in-house labeled using MaxPar labeling kits (Fluidigm Corporation) at UCB Pharma following the manufacturer's instructions, titrated and evaluated for binding to human PBMCs and/or control cell lines before use.

Supplemental Table 4. Primers cDNA synthesis

Oligo pool	Individual oligo	Sequence 5’-3’	Comment	Ref*
**i7-IgG/IgA-pool**	i7_IgA_H2016_rev	AGACGTGTGCTCTTCCGATCTNNNNNTNNNNTNNNGGGGAAGAAGCCCTGGAC	IgA antisense primer, CH1 1-24bp, with UMI and i7 adapter	1
	i7_IgG_H2016_rev	AGACGTGTGCTCTTCCGATCTNNNNNTNNNTNNNNAGTAGTCCTTGACCAGGCAG	IgG antisense primer, CH1 1-24bp, with UMI and i7 adapter	1
**i7-IgM/IgL-pool**	i7_UMI_IgM_rev	AGACGTGTGCTCTTCCGATCTNNNNTNNNNNTTNNNTAAGGGTTGGGGCGGATGCACTCCC	IgM antisense primer, CH1 1-24bp, with UMI and i7 adapter	N/A
	i7_UMI_KC_rev	AGACGTGTGCTCTTCCGATCTNNTNNNNTNNNNTNNTGAAGACAGATGGTGCAGCCACAGTTC	IgK antisense primer, 5' CK region with UMI and i7 adapter	N/A
	i7_UMI_LC_rev	AGACGTGTGCTCTTCCGATCTNNTNNNNNTNNTNNNAGTGACCGAGGGGTTGGCCTTGGGCTG	IgL antisense primer, 5' CL region with UMI and i7 adapter	N/A

1 Horns, F. *et al*. Lineage tracing of human B cells reveals the in vivo landscape of human antibody class switching. *Elife* **5**, doi:10.7554/eLife.16578 (2016).

* Oligo sequences were adapted from oligos used in the citation

Supplemental Table 5. Primers for PCR amplification from mRNA

Oligo pool	Individual oligo	Sequence 5'-3'	Comment	Ref*
**i5-VH pool**	i5-HV1_1_70_Fwrd	ACACTCTTTCCCTACACGACGCTCTTCCGATCTSCAGCTGGTGCAGTCTGG	IGHV1 forward primer, with i5 adapter attached	1
	i5-HV1/3/5_70_Fwrd	ACACTCTTTCCCTACACGACGCTCTTCCGATCTGTGCAGCTGGTGGAGTCTG	IGHV1/3/5 forward primer, with i5 adapter attached	1
	i5-HV2_Fwrd	ACACTCTTTCCCTACACGACGCTCTTCCGATCTTCACCTTGAAGGAGTCTGG	IGHV2 forward primer, with i5 adapter attached	1
	i5-HV4_1_Fwrd	ACACTCTTTCCCTACACGACGCTCTTCCGATCTTGCAGCTGCAGGAGTCG	IGHV4 forward primer, with i5 adapter attached	1
	i5-HV4_2_Fwrd	ACACTCTTTCCCTACACGACGCTCTTCCGATCTGTGCAGCTACAGCAGTGG	IGHV4 forward primer, with i5 adapter attached	1
	i5-HV6_Fwrd	ACACTCTTTCCCTACACGACGCTCTTCCGATCTGTACAGCTGCAGCAGTCA	IGHV6 forward primer, with i5 adapter attached	1
**i7-Rev**	i7-Rev	AGACGTGTGCTCTTCCGATCT	Antisense primer annealing to Illumina i7 adapter	Illumina P7

1 Horns, F. *et al*. Lineage tracing of human B cells reveals the in vivo landscape of human antibody class switching. *Elife* **5**, doi:10.7554/eLife.16578 (2016).

Supplemental Table 6. Primers Light chain PCR 1 amplification for mRNA libraries

Oligo pool	Individual oligo	Sequence 5'-3'	Comment	Ref*
**i5-VL pool**	HSCK1-F	GGGCCCAGGCGGCCGAGCTCCAGATGACCCAGTCTCC	IGKV/1 forward primer, with 5' extension adapter	2
	HSCK24-F	GGGCCCAGGCGGCCGAGCTCGTGATGACYCAGTCTCC	IGKV2/4 forward primer, with 5' extension adapter	2
	HSCK3-F	GGGCCCAGGCGGCCGAGCTCGTGWTGACRCAGTCTCC	IGKV3 forward primer, with 5' extension adapter	2
	HSCK5-F	GGGCCCAGGCGGCCGAGCTCACACTCACGCAGTCTCC	IGKV5 forward primer, with 5' extension adapter	2
	HSCLam1a	GGGCCCAGGCGGCCGAGCTCGTGBTGACGCAGCCGCCCTC	IGLV forward primer, with 5' extension adapter	2
	HSCLam1b	GGGCCCAGGCGGCCGAGCTCGTGCTGACTCAGCCACCCTC	IGLV forward primer, with 5' extension adapter	2
	HSCLam2	GGGCCCAGGCGGCCGAGCTCGCCCTGACTCAGCCTCCCTCCGT	IGLV forward primer, with 5' extension adapter	2
	HSCLam3	GGGCCCAGGCGGCCGAGCTCGAGCTGACTCAGCCACCCTCAGTGTC	IGLV forward primer, with 5' extension adapter	2
	HSCLam4	GGGCCCAGGCGGCCGAGCTCGTGCTGACTCAATCGCCCTC	IGLV forward primer, with 5' extension adapter	2
	HSCLam6	GGGCCCAGGCGGCCGAGCTCATGCTGACTCAGCCCCACTC	IGLV forward primer, with 5' extension adapter	2
	HSCLam78	GGGCCCAGGCGGCCGAGCTCGTGGTGACYCAGGAGCCMTC	IGLV forward primer, with 5' extension adapter	2
	HSCLam9	GGGCCCAGGCGGCCGAGCTCGTGCTGACTCAGCCACCTTC	IGLV forward primer, with 5' extension adapter	2
	HSCLam10	GGGCCCAGGCGGCCGAGCTCGGGCAGACTCAGCAGCTCTC	IGLV forward primer, with 5' extension	2
**i7-Rev**	i7-Rev	AGACGTGTGCTCTTCCGATCT	Antisense primer annealing to Illumina i7 adapter	Illumina P7

2 Barbas, C. F. I., Burton, D. R., Scott, J. K. & Silverman, G. J. *Phage Display: A Laboratory Manual*. (Cold Spring Harbor Laboratory Press, 2001).

Supplemental Table 7. Primers Light chain PCR 2 amplification for mRNA libraries

Individual oligo	Sequence 5'-3'	Comment	Ref*
i5-ext-fwrd	ACACTCTTTCCCTACACGACGCTCTTCCGATCTNNNNNGGGCCCAGGCGGCCGAGCTC	forward primer annealing to extension adapter, with i5 adapter	N/A
i7-Rev	AGACGTGTGCTCTTCCGATCT	antisense primer annealing to Illumina i7 adapter	Illumina P7

Supplemental Table 8. Illumina adapter primers

Index	Index sequence	primer	Oligo sequence
S502	CTCTCTAT	i5_indexS502_fwrd	AATGATACGGCGACCACCGAGATCTACACCTCTCTATACACTCTTTCCCTACACGACG
S503	TATCCTCT	255_i5_indexS503_fwrd	AATGATACGGCGACCACCGAGATCTACACTATCCTCTACACTCTTTCCCTACACGACG
S505	GTAAGGAG	257_i5_indexS505_fwrd	AATGATACGGCGACCACCGAGATCTACACGTAAGGAGACACTCTTTCCCTACACGACG
S506	ACTGCATA	258_i5_indexS506_fwrd	AATGATACGGCGACCACCGAGATCTACACACTGCATAACACTCTTTCCCTACACGACG
S507	AAGGAGTA	i5_indexS507_fwrd	AATGATACGGCGACCACCGAGATCTACACAAGGAGTAACACTCTTTCCCTACACGACG
S508	CTAAGCCT	i5_indexS508_fwrd	AATGATACGGCGACCACCGAGATCTACACCTAAGCCTACACTCTTTCCCTACACGACG
S510	CGTCTAAT	277_i5_indexS510_fwrd	AATGATACGGCGACCACCGAGATCTACACCGTCTAATACACTCTTTCCCTACACGACG
S511	TCTCTCCG	278_i5_indexS511_fwrd	AATGATACGGCGACCACCGAGATCTACACTCTCTCCGACACTCTTTCCCTACACGACG
N701	TCGCCTTA	i7_indexN701_rev	CAAGCAGAAGACGGCATACGAGATTCGCCTTAGTGACTGGAGTTCAGACGTGTGCTCTTCCGATCT
N702	CTAGTACG	i7_indexN702_rev	CAAGCAGAAGACGGCATACGAGATCTAGTACGGTGACTGGAGTTCAGACGTGTGCTCTTCCGATCT
N703	TTCTGCCT	271_i7_indexN703_rev	CAAGCAGAAGACGGCATACGAGATTTCTGCCTGTGACTGGAGTTCAGACGTGTGCTCTTCCGATCT
N704	GCTCAGGA	272_i7_indexN704_rev	CAAGCAGAAGACGGCATACGAGATGCTCAGGAGTGACTGGAGTTCAGACGTGTGCTCTTCCGATCT
N705	AGGAGTCC	i7_indexN705_rev	CAAGCAGAAGACGGCATACGAGATAGGAGTCCGTGACTGGAGTTCAGACGTGTGCTCTTCCGATCT
N706	CATGCCTA	i7_indexN706_rev	CAAGCAGAAGACGGCATACGAGATCATGCCTAGTGACTGGAGTTCAGACGTGTGCTCTTCCGATCT
N707	GTAGAGAG	273_i7_indexN707_rev	CAAGCAGAAGACGGCATACGAGATGTAGAGAGGTGACTGGAGTTCAGACGTGTGCTCTTCCGATCT
N710	CAGCCTCG	274_i7_indexN710_rev	CAAGCAGAAGACGGCATACGAGATCAGCCTCGGTGACTGGAGTTCAGACGTGTGCTCTTCCGATCT
N711	TGCCTCTT	i7_indexN711_rev	CAAGCAGAAGACGGCATACGAGATTGCCTCTTGTGACTGGAGTTCAGACGTGTGCTCTTCCGATCT
N712	TCCTCTAC	i7_indexN712_rev	CAAGCAGAAGACGGCATACGAGATTCCTCTACGTGACTGGAGTTCAGACGTGTGCTCTTCCGATCT
N714	TCATGAGC	275_i7_indexN714_rev	CAAGCAGAAGACGGCATACGAGATTCATGAGCGTGACTGGAGTTCAGACGTGTGCTCTTCCGATCT
N715	CCTGAGAT	276_i7_indexN715_rev	CAAGCAGAAGACGGCATACGAGATCCTGAGATGTGACTGGAGTTCAGACGTGTGCTCTTCCGATCT

Supplemental Table 9. Number of BCR sequences in NGS analysis

Subject	Group	mRNA reads	After UMI collaps	unique VH sequences*	unique VL sequences*
1	CCP positive RA	469156	207807	23430	11504
2	CCP positive RA	604724	377503	16973	10783
3	CCP positive RA	467126	140566	19701	6747
4	CCP positive RA	429050	267818	16078	6392
5	CCP positive RA	455023	310296	14098	8003
6	CCP positive RA	466422	260016	19236	8208
7	CCP positive RA	477797	339291	16528	9536
8	CCP positive RA	399470	281972	11970	6297
9	CCP positive RA	323087	207920	11554	4264
10	CCP positive RA	451009	317489	13537	5515
11	CCP positive RA	515217	204448	22137	4733
12	CCP positive RA	424484	329157	13527	5981
13	CCP positive RA	517531	257907	22108	6668
14	Healthy control	454985	274145	18998	5332
15	Healthy control	662178	358653	25175	9560
16	Healthy control	599481	265251	25210	4934
17	Healthy control	415966	167584	14419	2171
18	Healthy control	530602	325089	18091	5686
19	Healthy control	381553	185652	18677	2621

* Unique complete V(D)J variable region nucleotide sequences

Supplemental Table 10. Subjects included in the total IgA and IgM screening

IgA cohort

	**Population controls** (n=1300)	**All RA patients** (n=2166)			**ACPA negative RA** (n=730)		**ACPA positive RA** (n=1250)	
	Frequency/ Mean±SD [Median]	Frequency/ Mean±SD [Median]	p-value#	Adjusted p-value€	Frequency/ Mean±SD [Median]	p-value#	Frequency/ Mean±SD [Median]	p- value#
Age	52±11.8 [54]	50±12.8 [53]	0.0005		51±13 [55]	0.0003	50±12 [52]	0.004
Females	72% (935/1300)	71% (1552/2194)	NS (0.46)		71% (520/730)	NS (0.84)	71% (890/1250)	NS (0.71)
Smoking*	63% (810/1289)	69% (1474/2147)	0.0005		62% (453/724)	NS (0.94)	73% (910/1245)	<0.0001
HLA SE	51%(650/1285)	73% (1589/2166)	<0.0001		54% (393/721)	NS (0.10)	85% (1047/1236)	<0.0001
Total IgA (mg/ml)	1.58±0.80 [1.51]	2.32± 1.0 [2.21]	<0.0001	<0.0001	2.28±1.0 [2.15]	<0.0001	2.39±0.97 [2.25]	<0.0001

IgM cohort

	**Population controls** (n=154)	**All RA patients** (n=243)			**ACPA negative RA** (n=50)		**ACPA positive RA** (n=193)	
	Frequency/ Mean±SD [Median]	Frequency/ Mean±SD [Median]	p-value#	Adjusted p-value€	Frequency/ Mean±SD [Median]	p-value#	Frequency/ Mean±SD [Median]	p- value#
Age	53±10.7 [54]	49±12.3 [51]	0.002		50±13.7 [53]	NS (0.52)	49±11.9 [51]	0.002
Females	71% (110/154)	71% (174/243)	NS (1)		60% (30/50)	NS (0.16)	75% (144/193)	NS (0.54)
Smoking*	66% (100/152)	68% (166/242)	NS (0.58)		58% (29/50)	NS (0.40)	71% (137/192)	NS (0.29)
HLA SE	69%(106/154)	79% (192/242)	0.02		58% (29/50)	NS (0.17)	85% (163/192)	0.0004
Total IgM (mg/ml)	0.99±0.57 [0.88]	1.39±0.71 [1.22]	p<0.0001	p<0.0001	1.32±0.64 [1.1]	0.002	1.41±0.72 [1.24]	p<0.0001

* Ever smoking
# P-value from Kruskal-Wallis analysis compared with Dunn's multiple comparisons test or Fisher's exact test compared to controls
€ Nominal logistic regression model comparing IgM/IgA in all RA vs healthy controls, adjusting for age, sex, smoking and HLA shared epitope (SE)

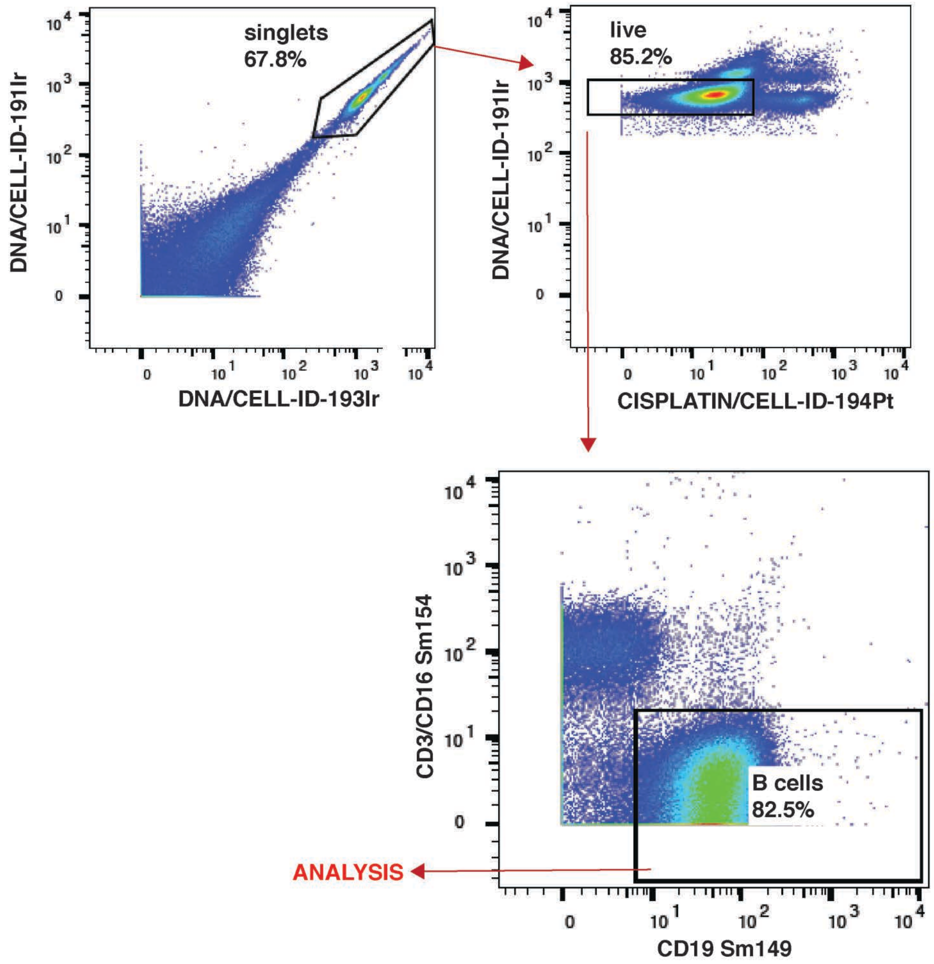

Supplemental Figure 1. Gating of mass cytometry data

Mass cytometry data was acquired on human PBMC samples enriched for B cells by MACS negative separation (B cells kit II, Miltenyi Biotec). The figure shows processing of the bead-normalized concatenated FCS files by manual gating in FlowJo (Treestar). The cells were gated for singlets by DNA staining, followed by exclusion of dead cells by cisplatin staining. B cells were gated as CD19+ CD3/CD16- cells. The exported files were used in subsequent bioinformatics analysis.

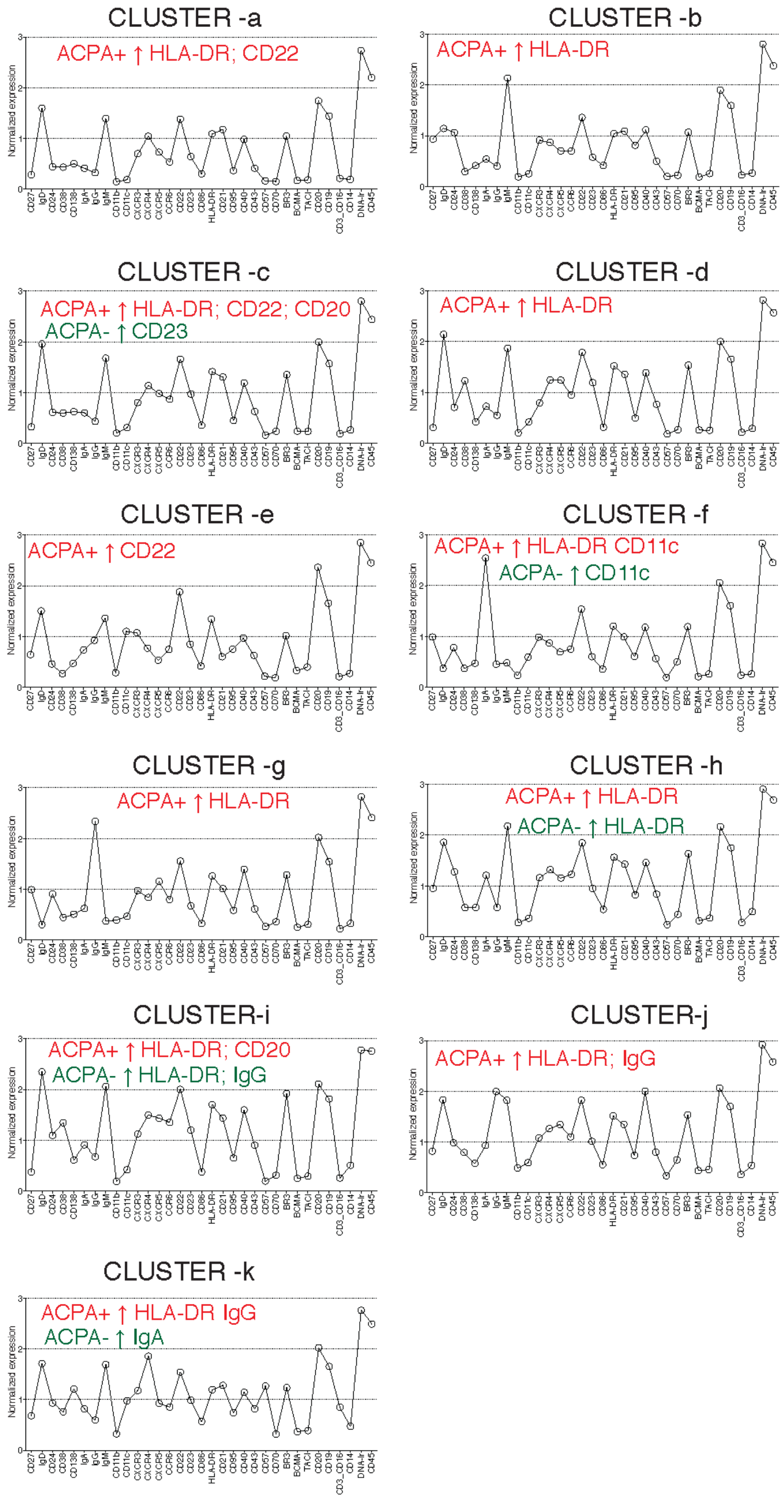

Supplemental Figure 2. B-cell mass cytometry expression profiles in cell clusters after clustering to identify differential expression

The figure visualizes the expression profiles of B-cell populations identified in the cluster analysis for differential expression analysis. The figure shows populations with significant differences between healthy controls (n=7), ACPA + RA (n=7) and ACPA- RA (n=9). Very small cell populations were excluded. Surface markers with statistically significantly differential expression (t-test, $p<0.05$) between either ACPA+ RA and healthy controls or ACPA- RA and healthy controls, are indicated in red or green text, respectively. Details are presented in Figure 1. All expression levels were normalized for visualization.

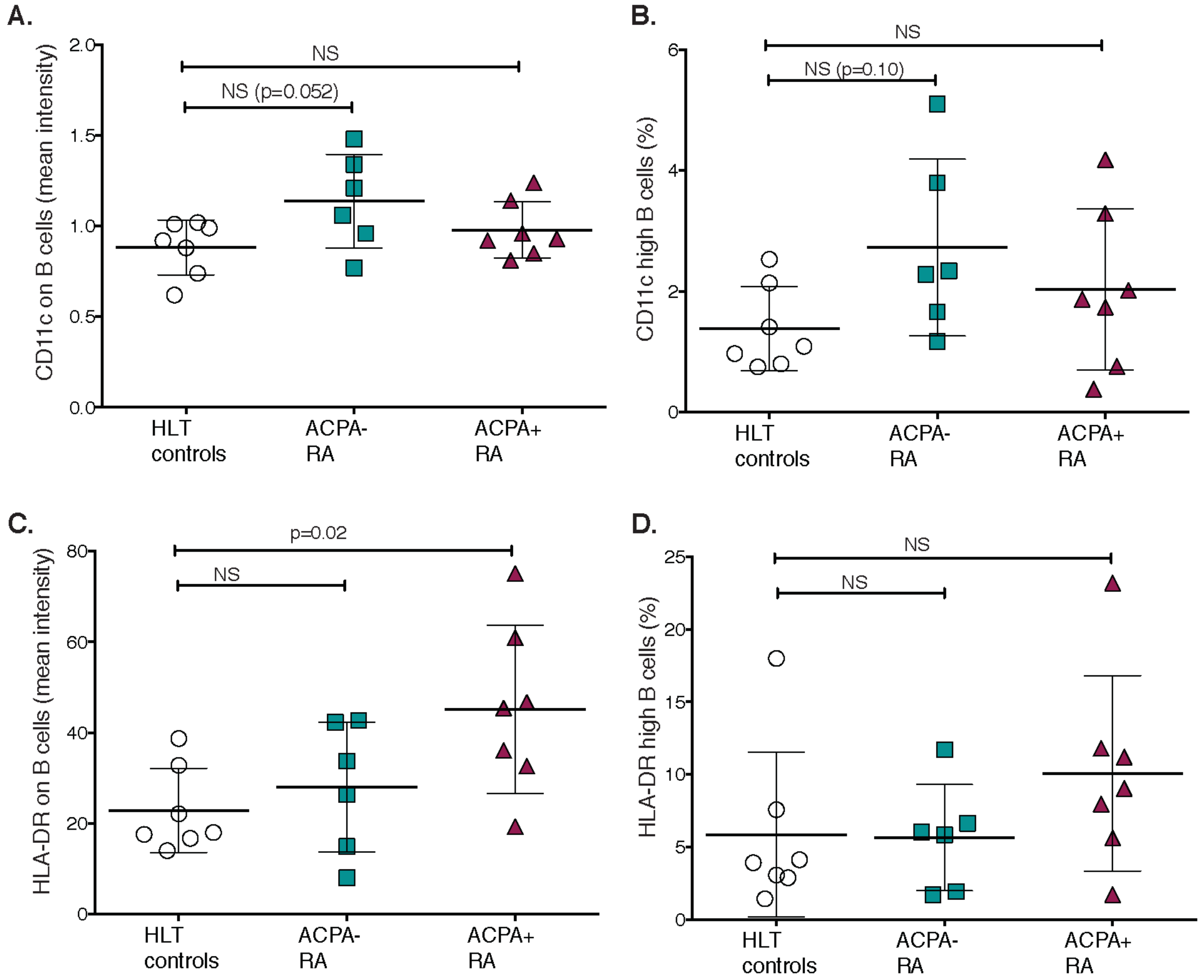

Supplemental Figure 3. Manual gating of B-cell mass cytometry data

Mass cytometry data was manually gated in FlowJo for validation of cluster analysis for differential expression. Samples with low cell counts were excluded from this analysis. CD11c (**A-B**) and HLA-DR (**C-D**) was evaluated on total B cells (CD19+ CD3/CD16-). P-values were derived from ANOVA analysis, adjusting for multiple comparisons.

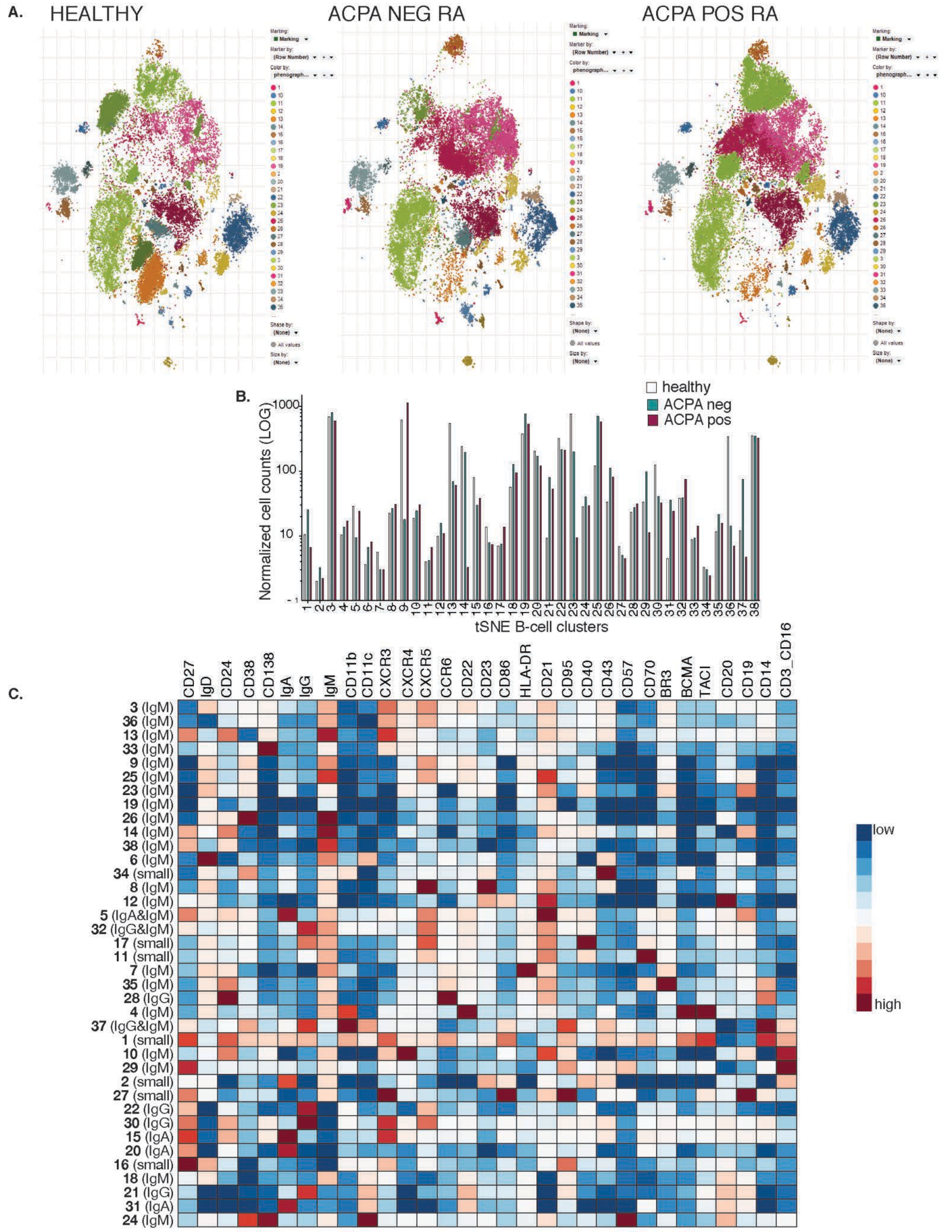

Supplemental Figure 4. B-cell mass cytometry cell counts in clustering and tSNE visualization

In order to explore differences in the size of B-cell populations between patient groups by relative cell counts, mass cytometry data was analyzed by tSNE (**A**) to depict different cell clusters, followed by ANOVA analysis to determined what cell populations that were significantly different between healthy controls, ACPA- RA and ACPA+ RA. The model adjusted for sex, age and cell processing date. **B**. Normalized cell counts for the different cell populations. **C**. Normalized expression profiles for the B-cell clusters identified in the cell count analysis. The heatmap visualize the expression in a color scale from red (high expression) to blue (low expression) compared to the other clusters. Small cell clusters were not included in the downstream analysis. Quantitative displays of the surface marker expression profiles for the clusters are shown in Supplemental Figure 5 and 7.

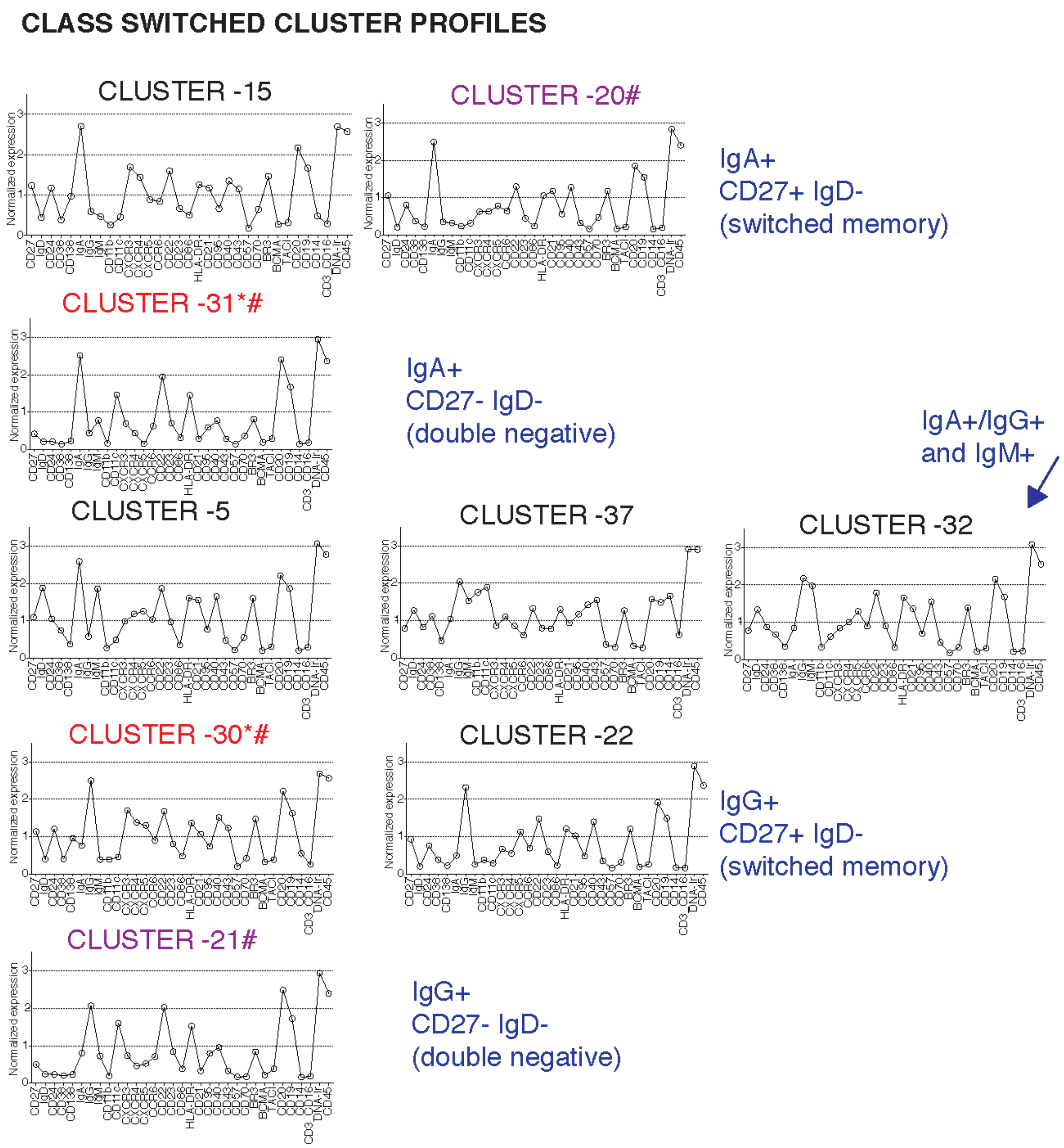

Supplemental Figure 5. B-cell mass cytometry expression profiles profiles of class-switched cell clusters after clustering for cell count analysis

The figure visualizes the expression profiles of class-switched (IgG+ or IgA+) B-cell populations identified in the tSNE analysis for cell population size (relative cell counts). Populations with significant ($p<0.05$) or clones to significant ($p=0.5-0.12$) differences between healthy controls ($n=7$), ACPA + RA ($n=7$) and ACPA- RA ($n=9$) are highlighted in red and with an asterisk (*). Clusters with a statistical difference when comparing only ACPA+ and healthy controls are highlighted in purple and (#). More details of these populations are shown in Figure 1. Cell counts are shown in Supplemental Figure 4. All expression levels were normalized for visualization.

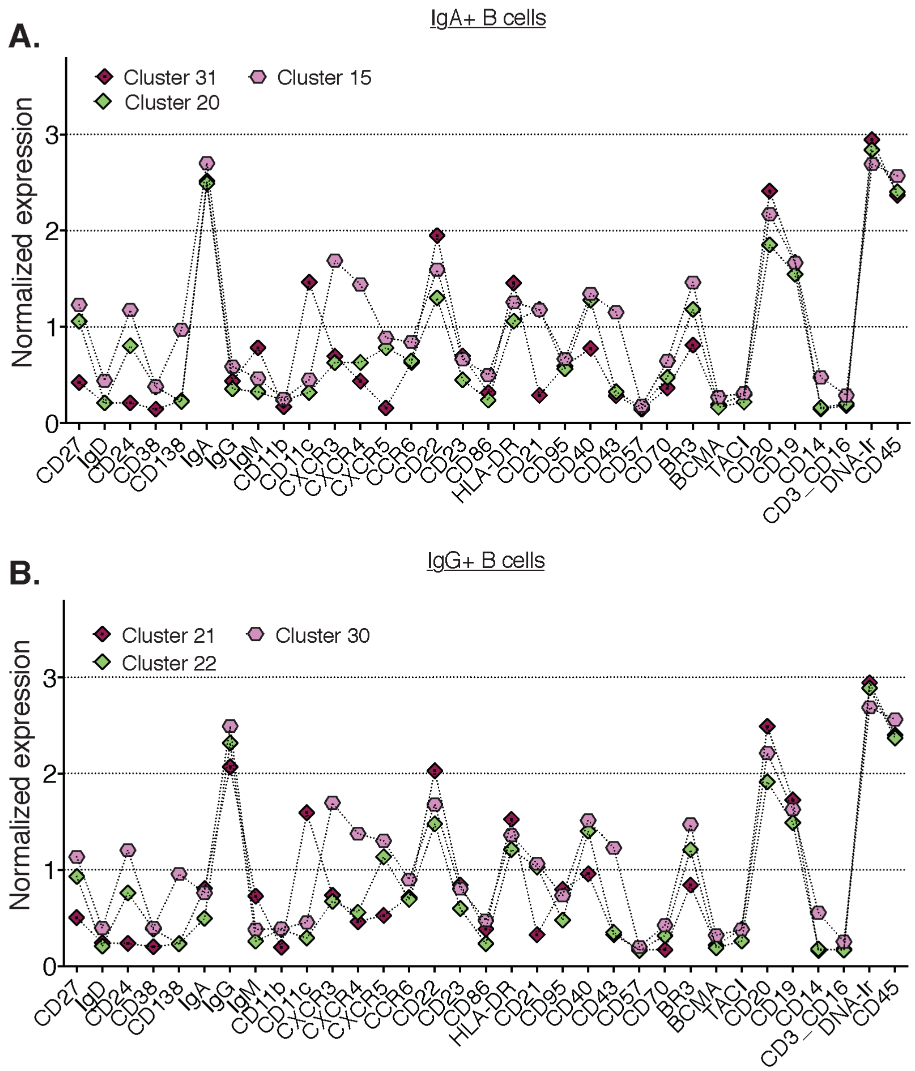

Supplemental Figure 6. Comparing expression profiles of class-switched cell clusters
The figure visualizes the expression profiles of class-switched (IgG+ or IgA+) B-cell populations identified in the tSNE analysis for cell population size (relative cell counts). Comparing expression profiles for **A**. IgA+ cells and **B**. IgG+ cells. All expression levels were normalized.

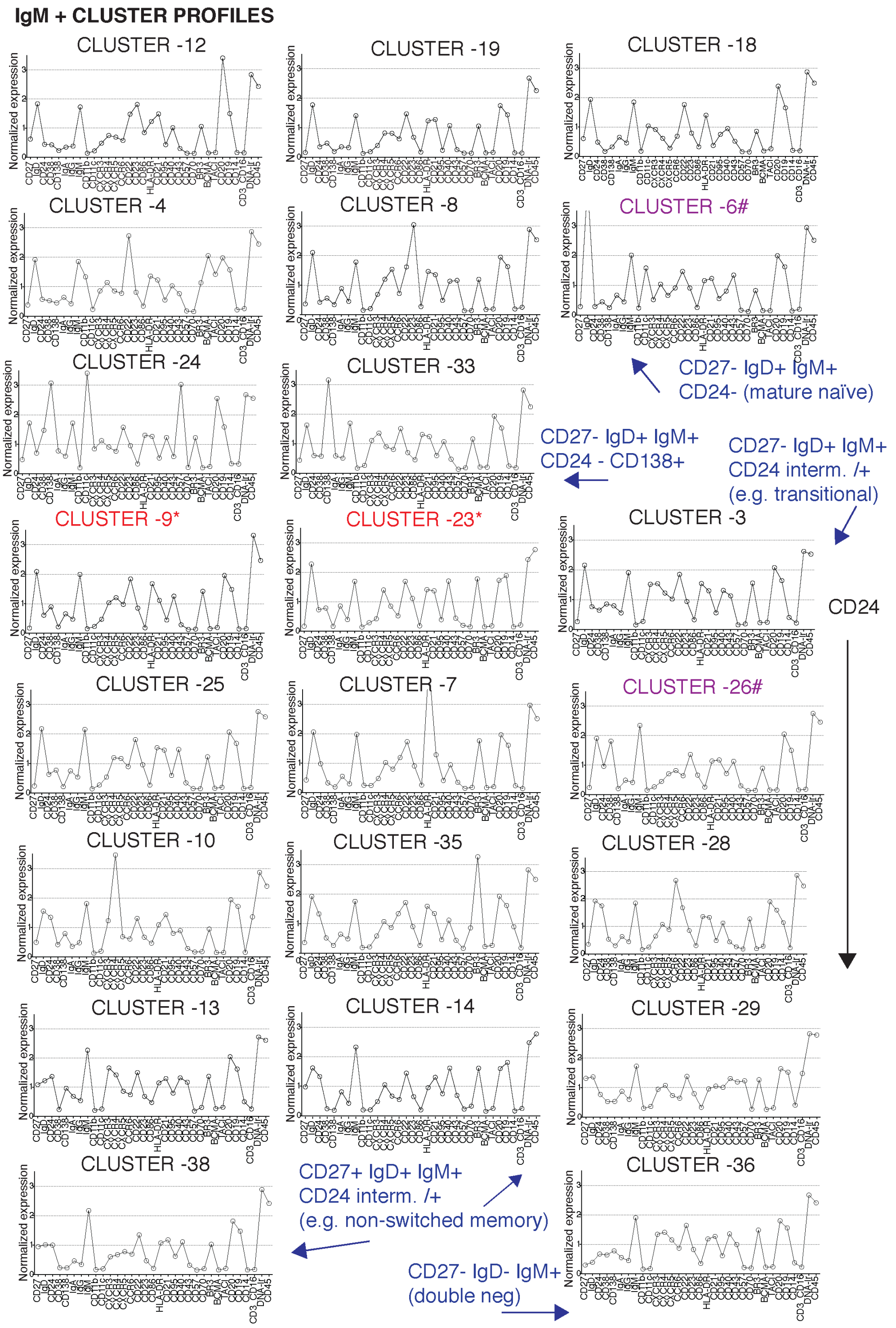

Supplemental Figure 7. B-cell mass cytometry expression profiles in IgM + cell clusters after clustering for cell count analysis

The figure visualizes the expression profiles of IgM+ B-cell populations identified by clustering and visualized in the tSNE analysis for cell population size (relative cell counts). Populations with significant ($p<0.05$) or clones to significant ($p=0.5-0.12$) differences between healthy controls (n=7), ACPA + RA (n=7) and ACPA- RA (n=9) are highlighted in red and with an asterisk (*). Clusters with a statistical difference when comparing only ACPA+ and healthy controls are highlighted in purple and (#). More details of these populations are shown in Figure 1. Cell counts are shown in Supplemental Figure 4. All expression levels were normalized for visualization.

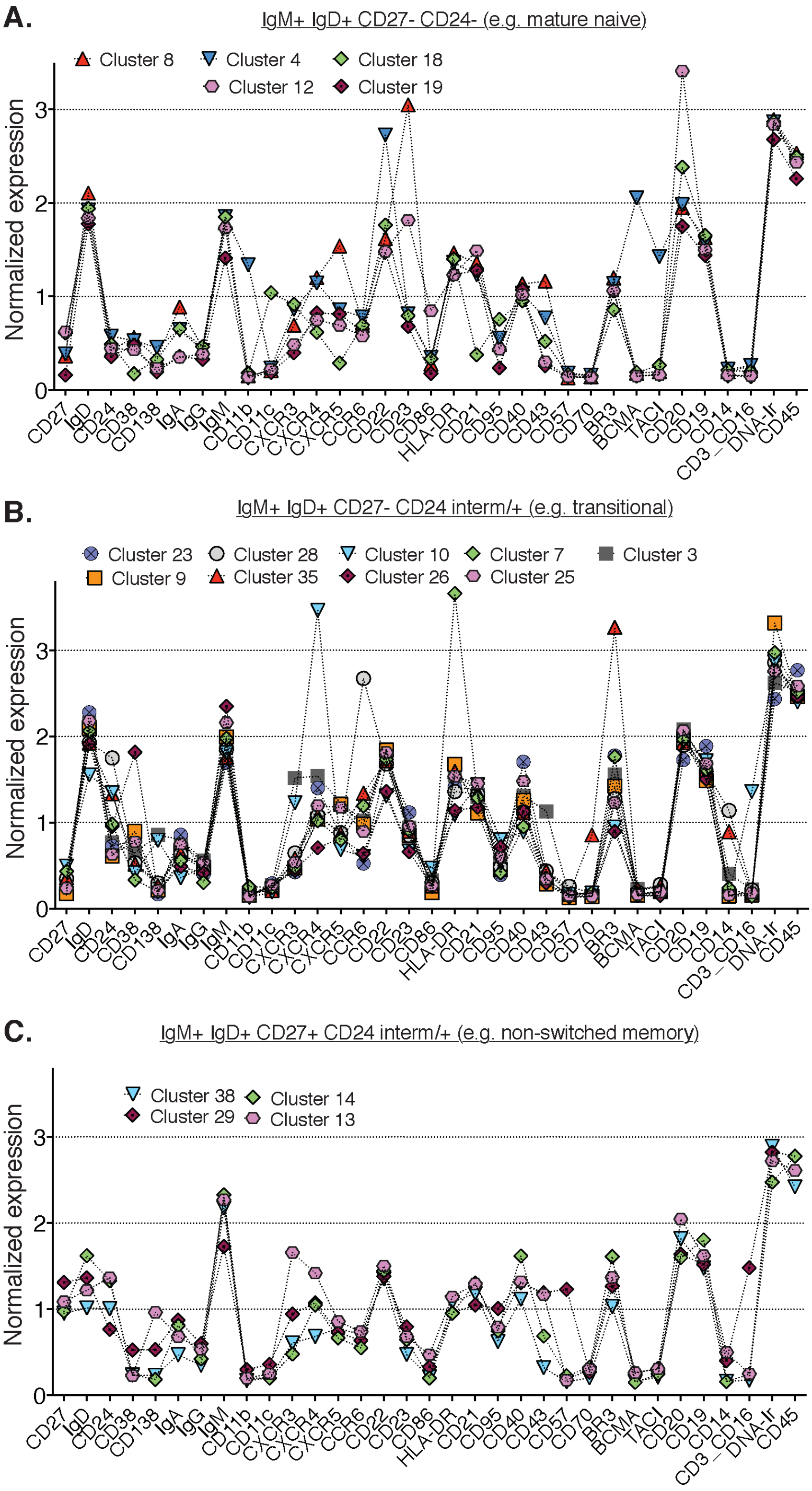

Supplemental Figure 8. Comparing expression profiles of IgM+ cell clusters
The figure visualizes the expression profiles of IgM+ B-cell populations identified in the tSNE analysis for cell population size (relative cell counts). Comparing expression profiles for **A**. IgM+ CD27-IgD+ CD24- cells **B**. IgM+ CD27-IgD+ CD24 intermediate or positive cells and **C**. IgM+ CD27+ IgD+ CD24 intermediate/positive cells. All expression levels were normalized.

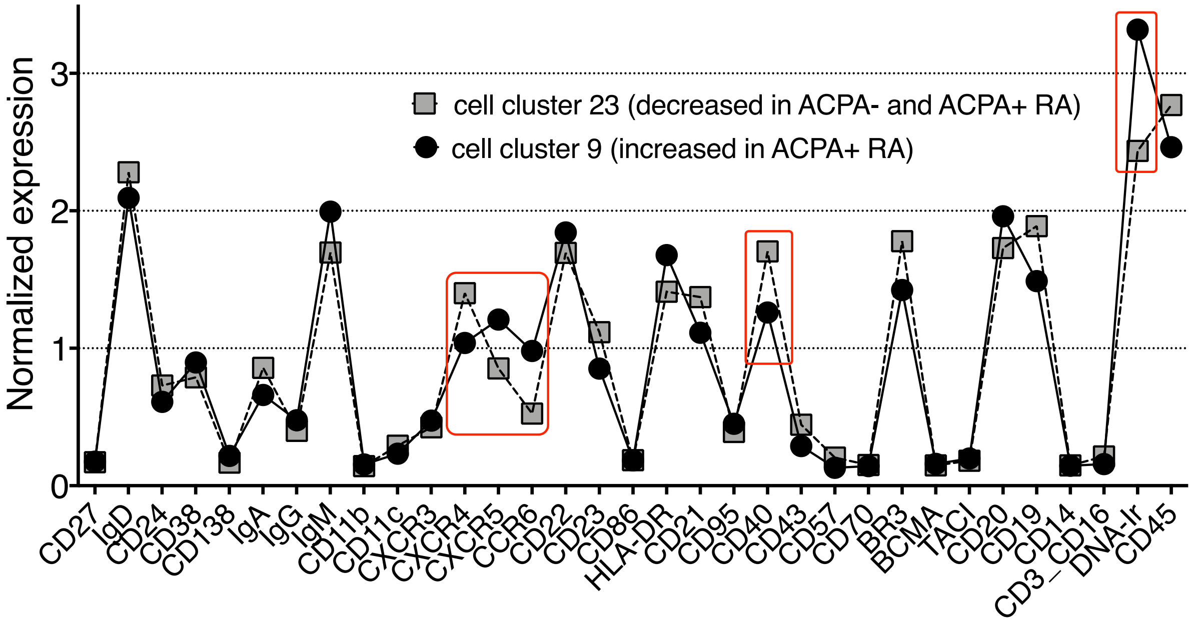

Supplemental Figure 9. B-cell mass cytometry tSNE clustering profiles in cell count analysis of two IgM+ clusters displaying different size in RA

The figure visualizes the expression profiles of two IgM+ B-cell populations identified in the tSNE analysis for cell population size (relative cell counts). Both clusters showed strong trends in the ANOVA analysis comparing healthy controls, ACPA+ RA and ACPA- RA (p=0.06). The cell counts for cell cluster 9 was higher in APCA+ RA (but low in ACPA- RA), while the opposite was seen for cell cluster 23 that was decreased in size in both ACPA+ and ACPA- RA compared to controls. Markers that seem to differ between these B cells populations are highlighted with red squares.

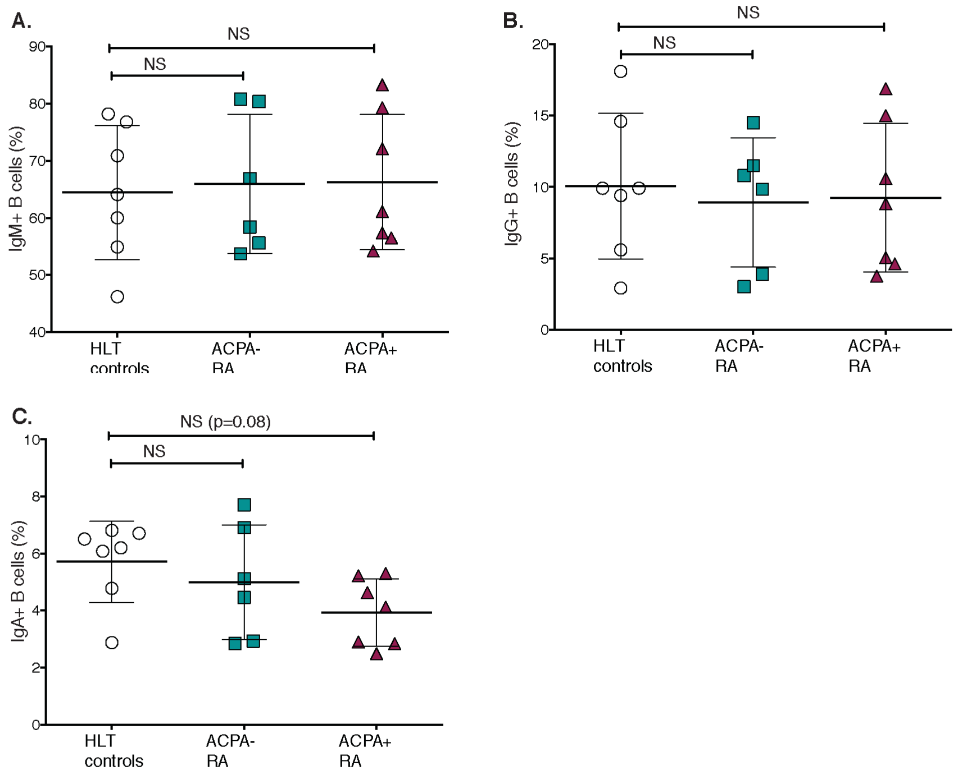

Supplemental Figure 10. Manual gating of B-cell mass cytometry data based on isotype positivity
Mass cytometry data was manually gated in FlowJo to explore differences in isotype (IgM, IgG or IgA) expression in B cells between healthy controls, ACPA- RA, and ACPA+ RA. Samples with low cell counts were excluded from this analysis. P-values were derived from ANOVA analysis, adjusting for multiple comparisons.

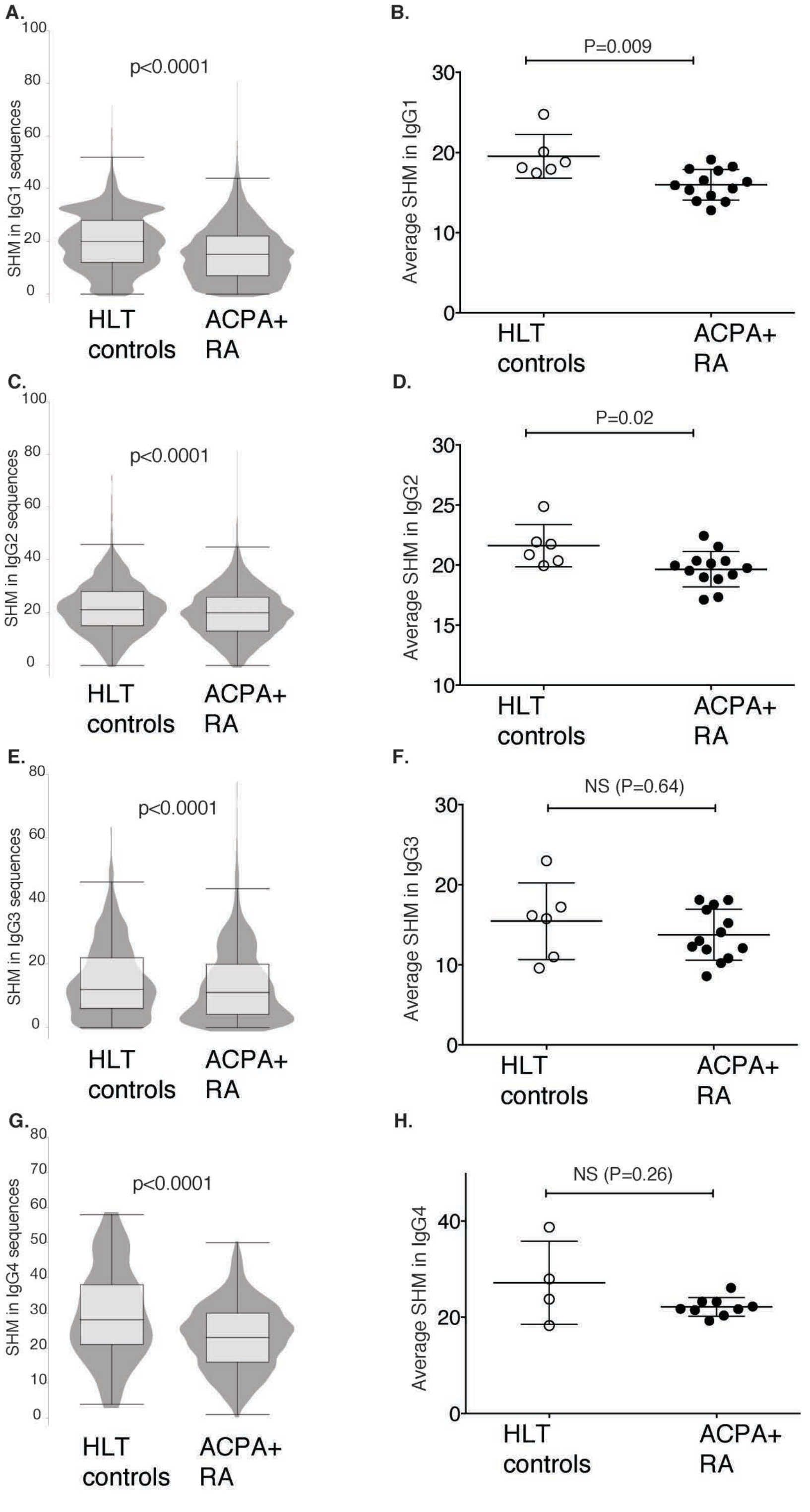

Supplemental Figure 11. Somatic hypermutation levels by NGS based on IgG subclass Results from llumina MiSeq of BCR from circulating B cells in ACPA+ RA compared to healthy controls. The level of SHM was determined by number of mismatches in comparison to the closest germline sequence in the IMGT database in IgG1 (**A-B**), IgG2 (**C-D**), IgG3 (**E-F**), and IgG4 (**G-H**) sequences. The left panels are showing result from pooled sequences (Turkey outliner box blots with an overlay violin plots), while the right panel are showing the average SHM in sequences from individual subjects. Notable, two healthy individuals were excluded in panel H due to few obtain sequences. P-values are presented from Mann-Whitney analysis.

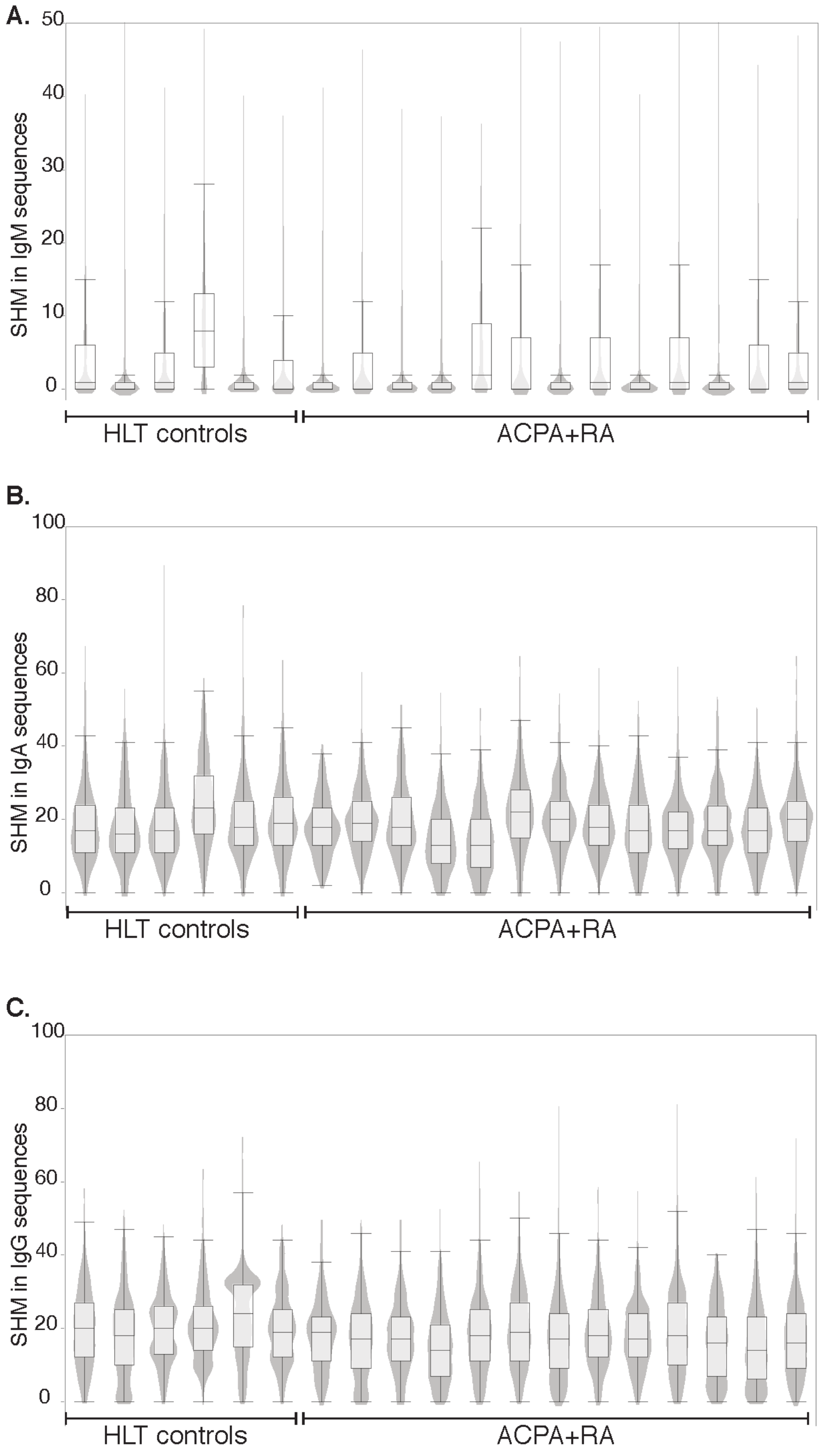

Supplemental Figure 12. Somatic hypermutation levels in individual samples analyzed by NGS

Results from llumina MiSeq of BCR from circulating B cells in ACPA+ RA compared to healthy controls. The level of SHM was determined by number of mismatches in comparison to the closest germline sequence in the IMGT database in IgM (**A**), IgA (**B**), and IgG (**C**) sequences. The distribution of SHM is shown by individual sample for six healthy subjects (HLT controls) and 13 ACPA+ RA patients (Turkey outliner box blots with overlay violin plots).

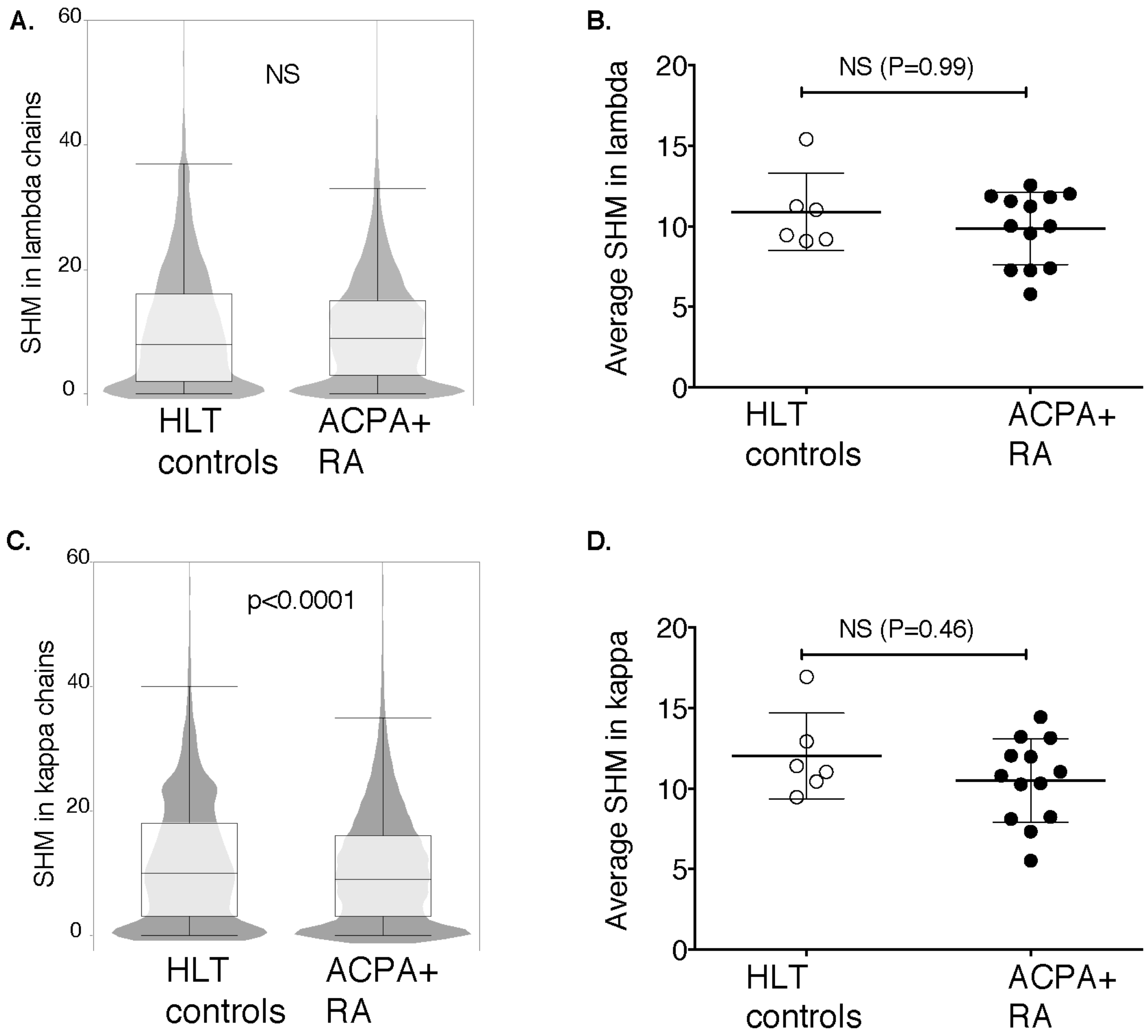

Supplemental Figure 13. Somatic hypermutation levels by NGS in light chains
Results from llumina MiSeq of VL BCR from circulating B cells in ACPA+ RA compared to healthy controls. The level of SHM was determined by number of mismatches in comparison to the closest germline sequence in the IMGT database in lambda (**A-B**) or kappa (**C-D** sequences. The left panels are showing result from pooled sequences (Turkey outliner box blots with an overlay violin plots), while the right panel are showing the average SHM in sequences from individual subjects. P-values are presented from Mann-Whitney analysis.

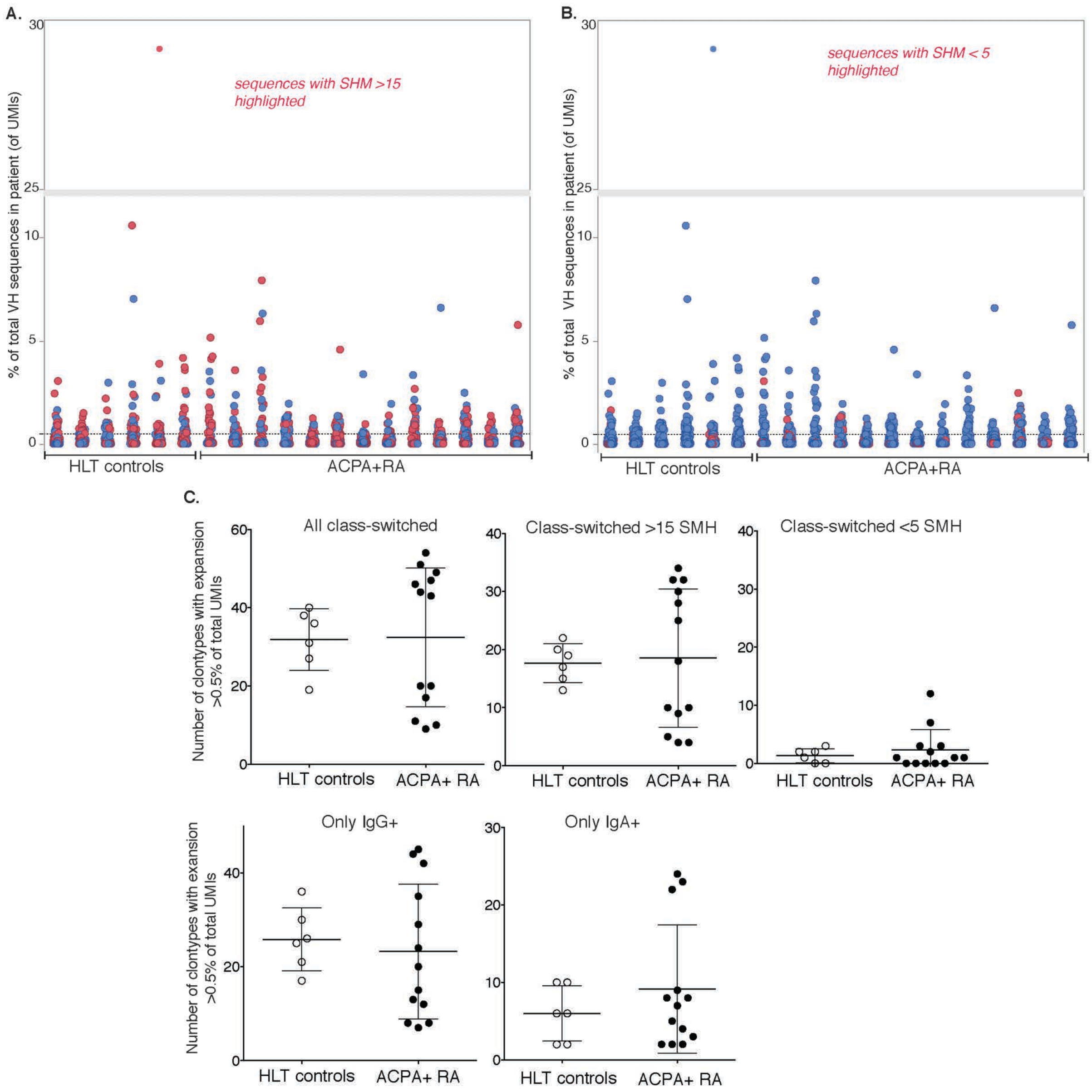

Supplemental Figure 14. Somatic hypermutation levels in expanded B-cell clones

Results from llumina MiSeq of VH BCR from circulating B cells in ACPA+ RA compared to healthy controls. An estimate of class-switched clonotype expansion was determined by unique molecular identifiers per clonotype (defined by unique VDJ rearrangement and CDR3 nucleotide sequence). The figure is showing the level of expansion of clonotypes (by % of all UMIs) per individual samples, highlighting (**A**) highly mutated sequences (IgG >15 SHM) in the clonotype reference or (**B**) unmutated/low SHM sequences (IgG <5 SHM) in red. The line at 0.5% indicate the cutoff for clones with expansion and/or high transcript levels. The number of expanded IgG/IgA clones per individual are depicted in (**C**). No significant difference between RA patients and healthy control in terms of number of expanded clones and the also healthy individuals show the presence of expansion, although two RA patient groups with and without a high number of expanded clones could be observed. Notably, the bulk sequencing would not be able to discriminate between B cells with increased transcript levels (plasmablasts) and expanded clones.

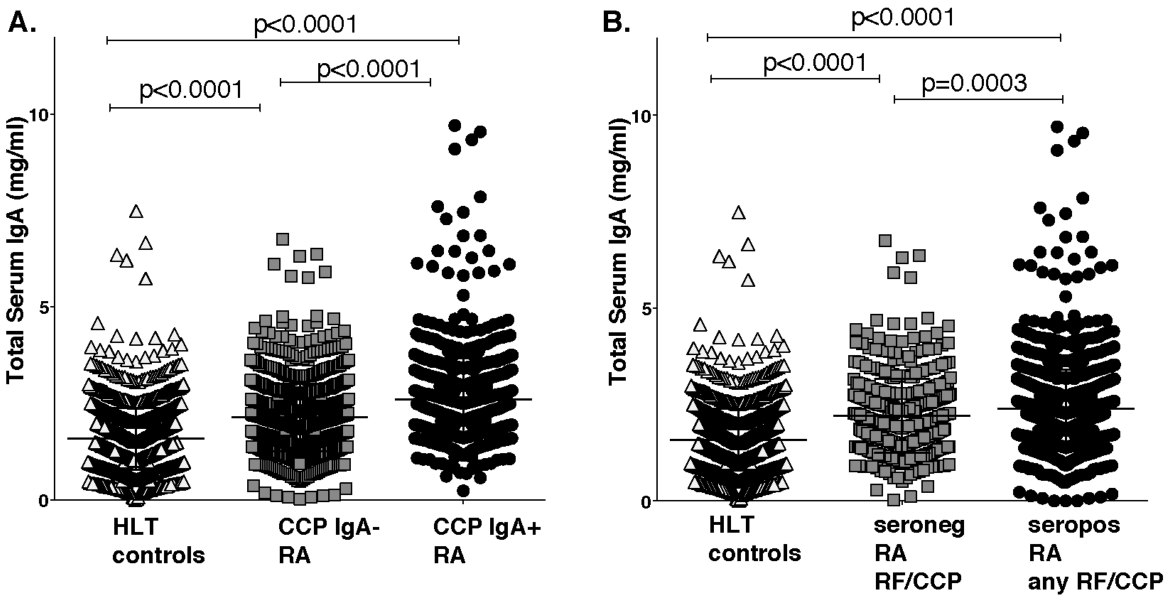

Supplemental Figure 15. Serum IgA levels in relation CCP2 and RF positivity

A. Total IgA in 1300 population controls, 1057 RA patients negative for CCP2 IgA and 920 RA patients positive for CCP2 IgA. **B**. Total IgA in 1300 controls and 465 seronegative RA patients without RF IgG, IgA, IgM or CCP IgG, IgA compared to 1517 seropositive RA patients with any positive autoantibody test. P-values are presented from Kruskal-Wallis analysis.
